# Host cellular protein RAB33B facilitates influenza viral replication and modulates M2 trafficking by enhancing autophagy

**DOI:** 10.1186/s13567-025-01560-6

**Published:** 2025-07-01

**Authors:** Shaotang Ye, Zhen Wang, Gang Lu, Aolei Chen, Liang Xu, Yongbo Liu, Jianwei Mao, Jingyu Wang, Gaoming Lou, Qingmei Xie, Kun Jia, Shoujun Li

**Affiliations:** 1 College of Veterinary Medicine, South China Agricultural University, Guangzhou, China; 2 Henry Fok School of Biology and Agriculture, Shaoguan University, Shaoguan, China; 3 Guangdong Provincial Key Laboratory of Utilization and Conservation of Food and Medicinal Resources in Northern Region, Shaoguan University, Shaoguan, China; 4 College of Animal Science, Hebei Normal University of Science & Technology, Qinhuangdao, China; 5 College of Animal Science, South China Agricultural University, Guangzhou, China

**Keywords:** IAV, M2 protein, autophagy, RAB33B, host–pathogen interaction, membrane trafficking

## Abstract

Influenza A virus (IAV) remains a major global health threat. Its M2 protein plays crucial roles in viral fusion, transportation, assembly, and release. Recent studies have shown that IAV impairs host autophagy flux to enhance viral replication. However, the precise mechanisms by which IAV M2 manipulates host cellular autophagy during virus replication remain unclear. In this study, we analysed cellular transcriptional responses of cells to IAV M2 overexpression and identified RAB GTPase protein RAB33B as a key factor. RAB33B was significantly up-regulated by IAV M2 and promoted IAV replication by enhancing autophagy. We further found that autophagy regulates the interaction of IAV M2, RAB33B, and LC3, facilitating M2 membrane trafficking through autophagic-like vesicles. In addition, ATG16L1 (an effector of RAB33B) and TBC1D25 (a GTPase-activating protein for RAB33B) contributed to IAV M2-induced autophagy, thereby affecting viral replication. Collectively, our findings reveal a novel mechanism in which RAB33B is essential for IAV M2 trafficking to the plasma membrane, facilitating viral replication through enhanced autophagy. These insights shed new light on the autophagy-based cellular transport mechanisms of IAV M2 and highlight potential antiviral targets.

**Supplementary Information:**

The online version contains supplementary material available at 10.1186/s13567-025-01560-6.

## Introduction

Influenza A virus (IAV) poses significant global health risks as a highly contagious respiratory pathogen. It is a single-stranded, negative-sense virus belonging to the family *Orthomyxoviridae* and is classified into 18 subtypes based on its hemagglutinin (HA) protein [1]. Waterfowl are currently recognised as the original host of IAV. Due to the HA protein’s ability to bind to two diverse sialic acid receptors, avian influenza viruses have evolved into a new potential threat to a wide range of mammalian species [2–4]. The IAV segments encode at least 10 proteins, including hemagglutinin (HA), neuraminidase (NA), matrix proteins 1 (M1) and 2 (M2), polymerase acidic (PA), polymerase basic 1 (PB1), polymerase basic 2 (PB2), nucleoprotein (NP), non-structural proteins 1 (NS1) and 2 (NS2). Among these, the M2 protein plays critical roles at various stages of viral infection in the host cells [5].

M2 comprises 97 amino acids and contains three structural domains: the N-terminal ectodomain, the transmembrane (TM) domain, and the C-terminal cytoplasmic tail. It functions as a proton-selective ion channel protein [6], facilitating viral entry, assembly, and budding. Consequently, M2 is considered an ideal target for developing antiviral drugs. A recent study has demonstrated that M2 undergoes ubiquitination and subsequent lysosome degradation [7]. However, M2 can counteract lysosomal degradation by inhibiting late-stage autophagy [8, 9]. Therefore, targeting the role of M2 in viral replication or its interaction with host-virus protein represents a promising strategy for developing novel antiviral drugs. The complex interplay between M2 and autophagy presents a valuable focus for future antiviral drug development.

Autophagy is a crucial cellular process that degrades damaged organelles and defends against pathogens. It plays a key role in cellular protection and the processing of antigens from foreign microorganisms [10]. Host cell autophagy processes rely on various signalling pathways and systems, including the PI3K-AKT-mTOR and AMPK pathways and the ATG12-ATG5-ATG16L1 and LC3B/ATG8 ubiquitin-like systems [11]. Previous studies have shown that host cells can degrade viral particles via autophagy, thereby inhibiting virus replication. However, many viruses have evolved diverse evasion strategies [12]. Following adaptation to mammalian cells, IAV can evade autophagic degradation of viral ribonucleoprotein complexes by weakening interactions with the P62 protein in mammal cells [13]. To date, most research on IAV-autophagy interactions has focused on how IAV viral proteins modulate the breakdown of autophagy [8, 9, 14] and inhibit the autophagic degradation of innate immune-related proteins [15–18]. These studies demonstrate that autophagy plays a dual role in IAV infection. Given this complexity, further investigation is urgently needed to understand how IAV regulates autophagy and how this regulation influences viral proteins, especially IAV M2, which triggers the activation of host cell autophagy.

Many proteins, such as RAB GTPase, play distinct roles in regulating autophagosome maturation and fusion. RAB1, RAB5 and RAB33B participate in autophagosome formation, while RAB7, RAB9A, RAB11, RAB23 and RAB32 are involved in autophagosome-lysosome fusion [19, 20]. The IAV M2 protein has been shown to employ RAB7 to restrict autolysosome formation and thereby evade degradation [9]. RAB GTPase serves as the coordinator for the intracellular vesicles in membrane trafficking [21]. Studies have shown that viruses, including IAV and coronaviruses, manipulate specific parts of RAB GTPase to promote viral protein trafficking, budding, and replication [22, 23]. However, it remains unclear whether IAV can hijack the autophagy-related RAB GTPase functions to support the membrane trafficking of viral proteins during replication.

Here, we used canine influenza virus (CIV) infection as a model to study IAV-induced autophagy and its effects on the M2 viral protein. We initially performed transcriptome analysis, which revealed that RAB33B is a host factor stimulated by CIV M2 and contributes to enhanced CIV replication. Our findings reveal a novel role for IAV-induced autophagy, wherein RAB33B, as a critical factor induced by IAV M2, facilitates IAV replication and M2 membrane trafficking. This process is mediated via autophagic mechanisms involving ATG16L1 and TBC1D25. Together, these results address critical gaps in our understanding of the relationship between IAV infection, host autophagy, and viral protein membrane trafficking.

## Materials and methods

### Cells, viruses, and plasmids

Madin-Darby canine kidney (MDCK) cells (CCL-34), human embryonic kidney (HEK) 293 T cells (CRL-3216) and human lung epithelial A549 cells (CCL-185) were obtained from American Type Culture Collection (Manassas, USA). HEK 293FT cells (JNO-H0487) were obtained from Guangzhou Jennio Biotech (Guangzhou, China). All cells were cultured at 37 °C in an atmosphere of 5% (v/v) CO_2_ in Dulbecco’s modified Eagle medium (DMEM; Biological Industries, Kibbutz Beit-Haemek, Israel) with 10% foetal bovine serum (FBS; Biological Industries). Subtypes of IAV used in this study included H1N1 (A/Puerto Rico/8/1934), H3N2 (A/canine/Guangdong/02/2011), and H5N1 (A/canine/Guangdong/01/2013). All virus strains were propagated in specific pathogen-free chick embryos. Plasmids used in this research are listed in Additional file 8. All virus-related protocols were approved by the ABSL-3 Committee of South China Agricultural University.

### Reagents and antibodies

The antibodies used in this study were as follows: IAV NP (GTX125989, 1:1000), IAV M2 (GTX125951, 1:1000), GAPDH (GTX100118, 1:5000), TBC1D25 (GTX132577, 1:1000), mCherry tag (GTX128508, 1:1000), HA tag (GTX115044, Rabbit, 1:1000), Myc tag (GTX115046, Rabbit, 1:1000), (GeneTex, Irvine, USA); RAB33B (Frontier Institute, AB2572278, 1:1000); ATG16L1 (AB187671, 1:1000), LC3 (AB192890, 1:2000), P62 (AB109012, 1:1000), Alexa Fluor^®^ 680 (AB175773, 1:5000), Alexa Fluor^®^ 790 (AB175781, 1:5000), Alexa Fluor^®^ 405 (AB175651, 1:1000), Alexa Fluor^®^ 488 (AB150113, 1:1000), Alexa Fluor^®^ 594 (AB150080, 1:1000), Alexa Fluor^®^ 647 (AB150115, 1:1000) (Abcam, Cambridge, UK); Myc tag (Santa Cruz Biotechnology, Dallas, USA; SC40, Mouse, 1:200); and HA tag (Beyotime Biotechnology, Shanghai, China; AH158, Mouse, 1:200).

The reagents used included rapamycin (1 μM, acting as an autophagy inducer, T1537), LY294002 (20 μM, acting as an autophagy inhibitor, T2008), and chloroquine (50 μM, acting as an autophagy inhibitor, T8689), doxycycline (4 μg/mL, T1687) (Topscience, Shanghai, China) and puromycin (5 μg/mL, Beyotime Biotechnology, ST551).

### Virus infection

For different experiments, cells were seeded at an appropriate density in culture plates and infected with IVA at a specific multiplicity of infection (MOI). Following incubation for 1 h at 37 °C, cells were rinsed thrice with phosphate-buffered saline (PBS). The culture medium was then replaced by fresh DMEM supplemented with FBS.

### Generation of MDCK/HEK293FT-TetOn-M2 cell line

MDCK and HEK293FT cells were transfected with TetOn-EGFP-M2 (based on the tetracycline-inducible expression system) using Lipo8000 (Beyotime Biotechnology, C0533), respectively. After 48 h of transfection, puromycin was added to the medium for selection. Positive cells were propagated for subsequent experiments. Doxycycline was used as the inducer to achieve stable and continuous overexpression of CIV M2. The results are presented in Additional file 14 to Additional file 16.

### Quantitative PCR

Quantitative polymerase chain reaction (qPCR) was performed using a LightCycler 480 system (Roche, Basel, Switzerland) with ChamQ SYBR qPCR Master Mix (Vazyme Biotechnology, Nanjing, China), following the manufacturer’s protocol. Relative mRNA expression levels were calculated using the 2^−∆∆Ct^ method, which indicated a fold change compared to the control group. Primer sets used for qPCR were designed based on published sequences and are listed in Additional file 9.

### RNA interference

The indicated small interfering RNA (siRNA) and a scrambled negative control siRNA were synthesised by RiboBio (Guangzhou, China). The sequences of all siRNAs are provided in Additional file 10. HEK293T or MDCK cells were transfected with the siRNAs using Lipo8000 (Beyotime Biotechnology, C0533) following the manufacturer’s protocol. Gene silencing efficiency was assessed by qPCR.

### Western blotting

Cellular protein lysates were prepared using cell lysis buffer for western blot and immunoprecipitation (IP; Beyotime Biotechnology, P0013). Lysates were collected and centrifuged at 15 000 rpm for 15 min. A total of 30 μg of each sample was separated by SDS-PAGE and transferred onto a polyvinylidene difluoride (PVDF) membrane. The transferred membranes were blocked at room temperature for 10 to 30 min with QuickBlock Blocking Buffer for western blotting (Beyotime Biotechnology, P0252), then incubated overnight at 4 °C with the appropriate primary antibodies. After three washes with phosphate-buffered saline with Tween (PBST), secondary antibodies were incubated for 1 h at room temperature. Protein bands were visualised using the Odyssey Sa imaging system (Li-COR, Lincoln, USA).

### Co-immunoprecipitation

Cellular protein lysates were harvested as described above. Lysates were incubated with protein A/G beads with HA tag antibody (Beyotime Biotechnology, P2121) for 4 h at 4 °C or overnight. Following incubation, the beads were washed five times with cell lysis buffer for western blotting and IP. The resulting precipitates were analysed by standard western blotting.

### Immunogold transmission electron microscopy

Cell samples were fixed using the same protocol as the sample preparation with a transmission electron microscope. After washing with double-distilled water (ddH2O), cells were placed in glycine solution for 1 h at room temperature. Cells were then rinsed with ddH2O, dehydrated through a graded ethanol series (30% to 100%), and embedded in LR White resin. Slicing was performed using a nickel mesh on the ultramicrotome. Slices were incubated with a rabbit IAV M2 antibody, followed by 10 nm colloidal gold-conjugated goat anti-rabbit IgG and stained with 1% lead citrate. The prepared samples were observed using a transmission electron microscope (Hitachi TEM system, Tokyo, Japan; HT7800).

### Confocal microscopy observation

For indirect immunofluorescence analysis, cells were washed thrice with PBS and fixed with cold 4% paraformaldehyde at room temperature for 10 to 15 min. Cells were then blocked and permeabilised for 10 min at room temperature using QuickBlock Blocking Buffer for Immunol Staining (Beyotime Biotechnology, P0260).

Following blocking, cells were incubated overnight at 4 °C with the indicated primary antibody, then for 1 h at room temperature with the corresponding secondary antibody. Nuclei were stained with DAPI (Beyotime Biotechnology, C1002) for 5 min. Cell membranes were stained using Wheat Germ Agglutinin (WGA) Alexa Fluor 594 (Thermo Fisher Scientific, Waltham, USA; W11262, 2 μg/mL) for 10 min. Fluorescence signals were observed using a confocal laser scanning microscope (Leica Microsystems SP8 TCS; Leica Microsystems, Wetzlar, Germany). Three-dimensional image reconstruction was performed using LAS X software (Leica Microsystems). For live-cell imaging, unfixed cells were directly imaged using the same confocal laser scanning microscope.

### Next-generation sequencing analysis

Cellular RNA samples were isolated from HEK293T cells transfected with 2500 ng M2-Myc or 2500 ng empty vectors. Complementary DNA (cDNA) libraries were constructed using the NEBNext Ultra Directional RNA Library Prep Kit (New England Biolabs, Ipswich, USA; E7420), the NEBNext Poly(A)mRNA Magnetic Isolation Module (New England Biolabs, E7490), NEBNext Multiplex Oligos (New England Biolabs; E6444) according to the manufacturer’s instructions. The purified and enriched cDNA libraries were quantified by Agilent 2200 (Agilent Technologies, Santa Clara, USA), and the tagged cDNA libraries were pooled in equal ratios and used for 150 bp paired-end sequencing in a single lane of the Illumina HiSeq X Ten (Illumina, San Diego, USA). Adaptor sequences and low-quality reads were removed, with clean reads used for subsequent analysis. Unique reads were analysed to identify differentially expressed genes (DEGs) using the EdgeR package standard, log_2_ (fold change [FC]) > 1 or <  −1 and false discovery rate (FDR) < 0.05. To analyse the DEGs, Fisher’s exact test was conducted for the Gene Ontology (GO) analysis across three categories: molecular function, cellular component, and biological process. The significantly differential GO terms (*p* < 0.01) were also performed as GO trees. In addition, Fisher’s exact test was used for the Kyoto Encyclopedia of Genes and Genomes (KEGG) signalling pathways analysis. The DEGs identified from HEK293T cells transfected with 2500 ng M2-Myc or 2500 ng empty vectors are listed in Additional file 11. The raw data of CIV-infected MDCK cells were obtained from reference [32] and analysed as above.

### Statistical analysis

All data were analysed using the unpaired Student’s *t*-test with Prism Software v10.2 (GraphPad, San Diego, USA). (mean ± SD, * *p* < 0.05, ** *p* < 0.01, *** *p* < 0.001).

## Results

### Autophagy is regulated by CIV infection in host cells

**Figure 1:**
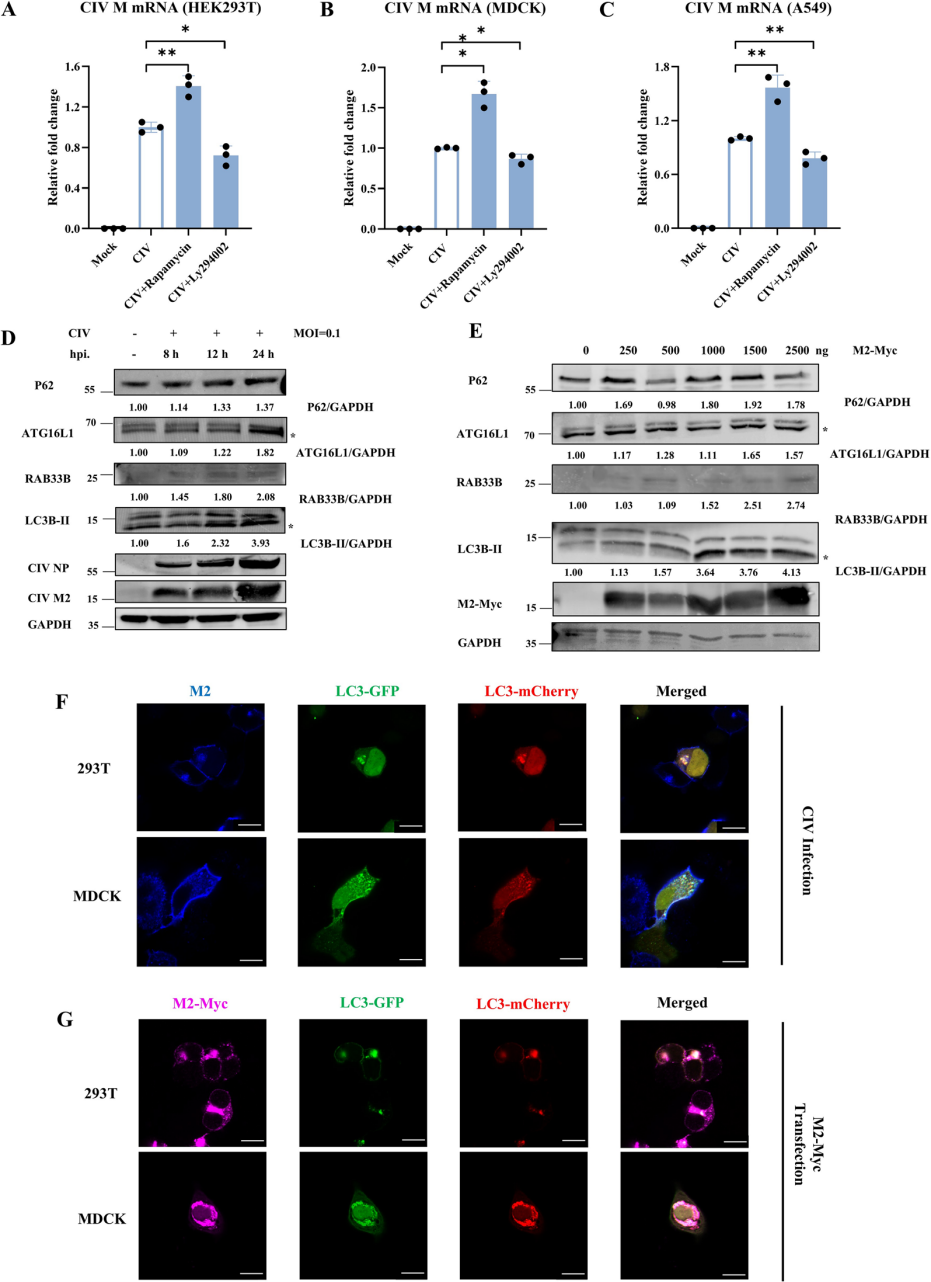
**CIV infection induces autophagy. A** CIV infection was regulated by the autophagy-modulation drug rapamycin and LY294002 in HEK293T cells, respectively. **B** CIV infection was regulated by autophagy-modulation drug rapamycin and LY294002 in MDCK cells, respectively. **C** CIV infection was regulated by autophagy-modulation drug rapamycin and LY294002 in A549 cells, respectively. **D** HEK293T cells were infected with CIV at MOI = 0.1 for 24 h, and cell lysates were analysed using western blotting. * represents the indicated protein. **E** HEK293T cells were transfected with different doses of CIV M2 plasmids. Cell lysates were analysed by western blotting. * represents the indicated protein. **F** HEK293T and MDCK cells were transfected with LC3-GFP-mCherry plasmid for 24 h and then infected with CIV at MOI = 0.1 for 24 h and analysed for the co-localisation of CIV M2 and LC3. Scale bar: 10 μm. **G** HEK293T and MDCK cells were co-transfected with CIV M2 and LC3-GFP-mCherry plasmids for 24 h, respectively, and analysed for the co-localisation of CIV M2 and LC3. Scale bar: 10 μm.

Previous studies have shown that IAV induces autophagy during infection and subsequently inhibits autophagic degradation to evade host immune defences. Furthermore, IAV proteins can selectively degrade host antiviral proteins via autophagy [9, 17, 24]. This interplay between autophagy and IAV is considered critical for viral replication. However, whether a similar mechanism is employed between CIV and host cell autophagy remains unclear. To elucidate this, we investigated whether autophagy induction could enhance intracellular CIV replication. We treated influenza virus-infected cells (HEK293T, MDCK, and A549) with an autophagy inducer (rapamycin) and an inhibitor (LY294002), respectively (Figures 1A–C). Cells were inoculated with CIV, and the CIV M gene content was measured using quantitative PCR. Our results showed that rapamycin treatment significantly increased CIV replication while LY294002 suppressed it. These findings suggest that CIV may utilise autophagy in a manner similar to other IAV subtypes.

To further assess the effect of CIV on host autophagy, we measured autophagy-related gene expression by western blotting. In CIV-infected cells, levels of ATG16L1, LC3B-II, and P62 protein expression were significantly increased (Figure 1D). Additionally, confocal microscopy revealed co-localisation of CIV M2 and LC3 within the cytoplasm and at the inner plasma membrane of HEK293T and MDCK cells (Figure 1F). Overexpression of the M2 protein produced autophagy-related effects comparable to those observed during CIV infection (Figures 1E and G). These results are consistent with prior studies indicating that the IAV M2 protein initiates autophagy, in part by redistributing LC3 to the plasma membrane [8, 14]. Collectively, our data indicate that CIV infection had a similar impact on host cell autophagy as human and avian IAV. Accordingly, we would use CIV to represent IAVs in further experiments.

### CIV M2 protein plays a crucial role in up-regulating the expression of gene *RAB33B*

**Figure 2:**
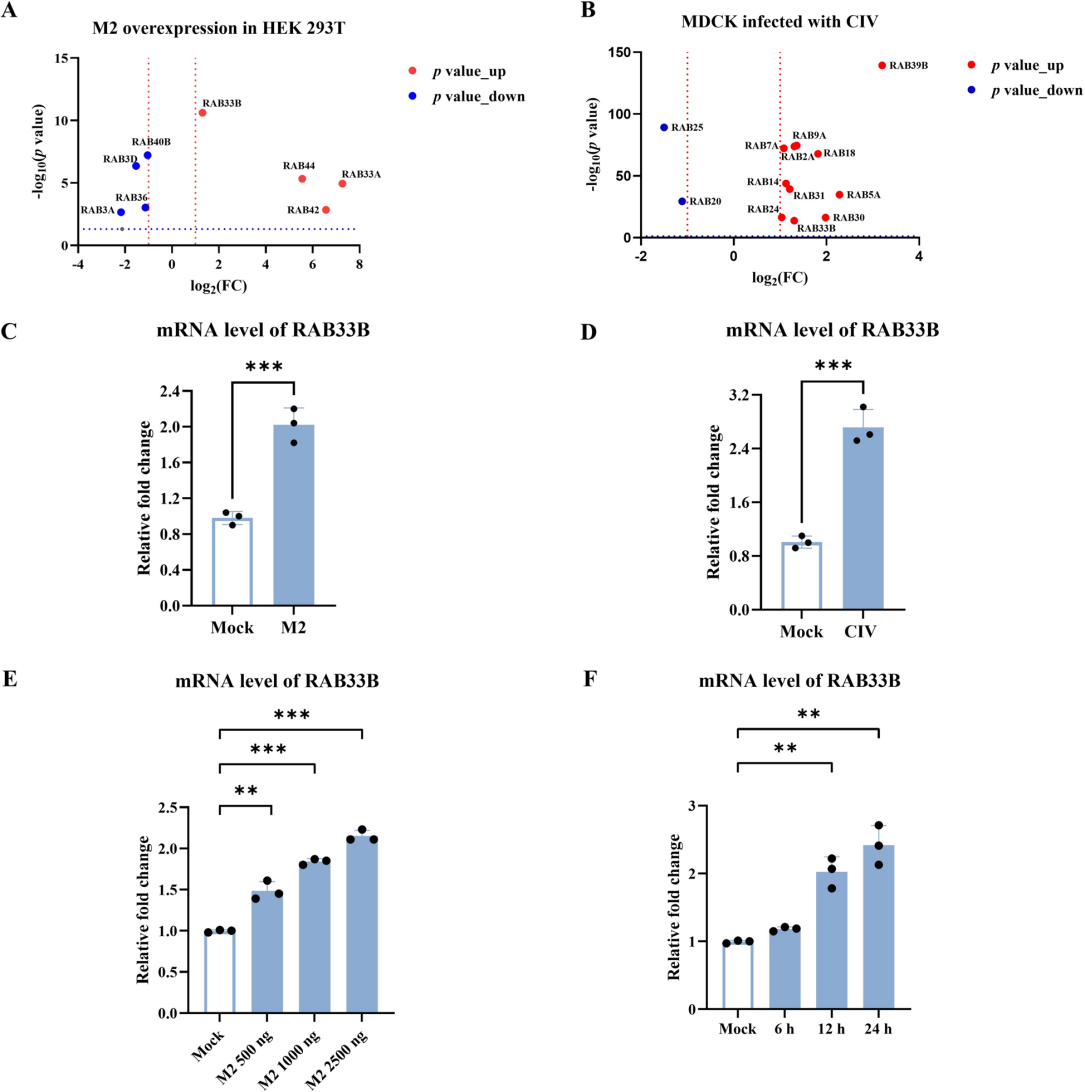
**CIV M2 protein regulates the expression of RAB33B. A** Volcano plot visualisation of DEGs of RAB genes in HEK293T cells transfected with the CIV M2 plasmid. **B** Volcano plot visualisation of DEGs of RAB genes in MDCK cells infected with CIV. **C** The relative mRNA expression levels of RAB33B were verified for **A**. **D** The relative mRNA expression levels of RAB33B were verified for **B**. **E** HEK293T cells were transfected with different doses of CIV M2 plasmids for 24 h, and the relative mRNA expression levels of RAB33B were tested. **F** HEK293T cells were infected with CIV M2 at MOI = 0.1 for 24 h, and the relative mRNA expression levels of RAB33B were detected.

Current research on IAV and autophagy has primarily focused on the M2 protein’s role in inhibiting lysosomal degradation during late-stage autophagy and how IAV proteins degrade the host’s innate immune-related proteins via selective autophagy. To understand the effect of CIV M2 on host cells, we performed transcriptome analysis of HEK293T cells transfected with CIV M2. This analysis identified 2811 DEGs in CIV M2-overexpressing cells, including 1398 up-regulated and 1413 down-regulated (Additional files 1A and 1B). Further analysis indicated that CIV M2 regulated multiple cell functions, including those related to membrane components and lysosomes (Additional file 1C). One study shows that IAV M2 disrupts interactions between RAB7 and TBC1D5, thereby preventing autolysosome formation and impairing viral component degradation [9]. However, the involvement of other RAB GTPase proteins in IAV-induced autophagy remains unclear. To address this, we examined transcriptome analysis and found that CIV M2 overexpression in HEK293T cells significantly up-regulated *RAB33A*, *RAB33B*, *RAB42*, and *RAB44* genes and down-regulated *RAB3A*, *RAB3D*, *RAB36*, and *RAB40B* genes (Figure 2A).

We further re-analysed transcriptome data from CIV-infected MDCK cells obtained in a previous study [32]. Our analysis showed a significant increase in *RAB33B* gene expression in MDCK cells with CIV infection (Figure 2B). We performed qPCR on both transcriptome data sets to confirm this finding, verifying *RAB33B* gene up-regulation (Figures 2C and D). Furthermore, to explore the relationship between CIV M2 and *RAB33B* gene expression in more detail, we performed gradient dose transfection and detected the time-different durations of infection in HEK293T. The results showed that *RAB33B* gene expression was positively regulated by CIV M2 overexpression in both a dose-dependent manner (Figures 1E and 2E) and in response to infection duration (Figures 1D and 2F).

In addition, HEK293FT and MDCK cell lines stably expressing M2-GFP were generated using a tetracycline-inducible expression system. Western blotting demonstrated a marked elevation in RAB33B protein expression following stable induction of M2-GFP protein (Additional files 14A and 14B). These findings suggested that the CIV M2 protein promotes *RAB33B* gene expression during IAV infection.

### The CIV replication is positively regulated by RAB33B through its pro-autophagy effect

**Figure 3:**
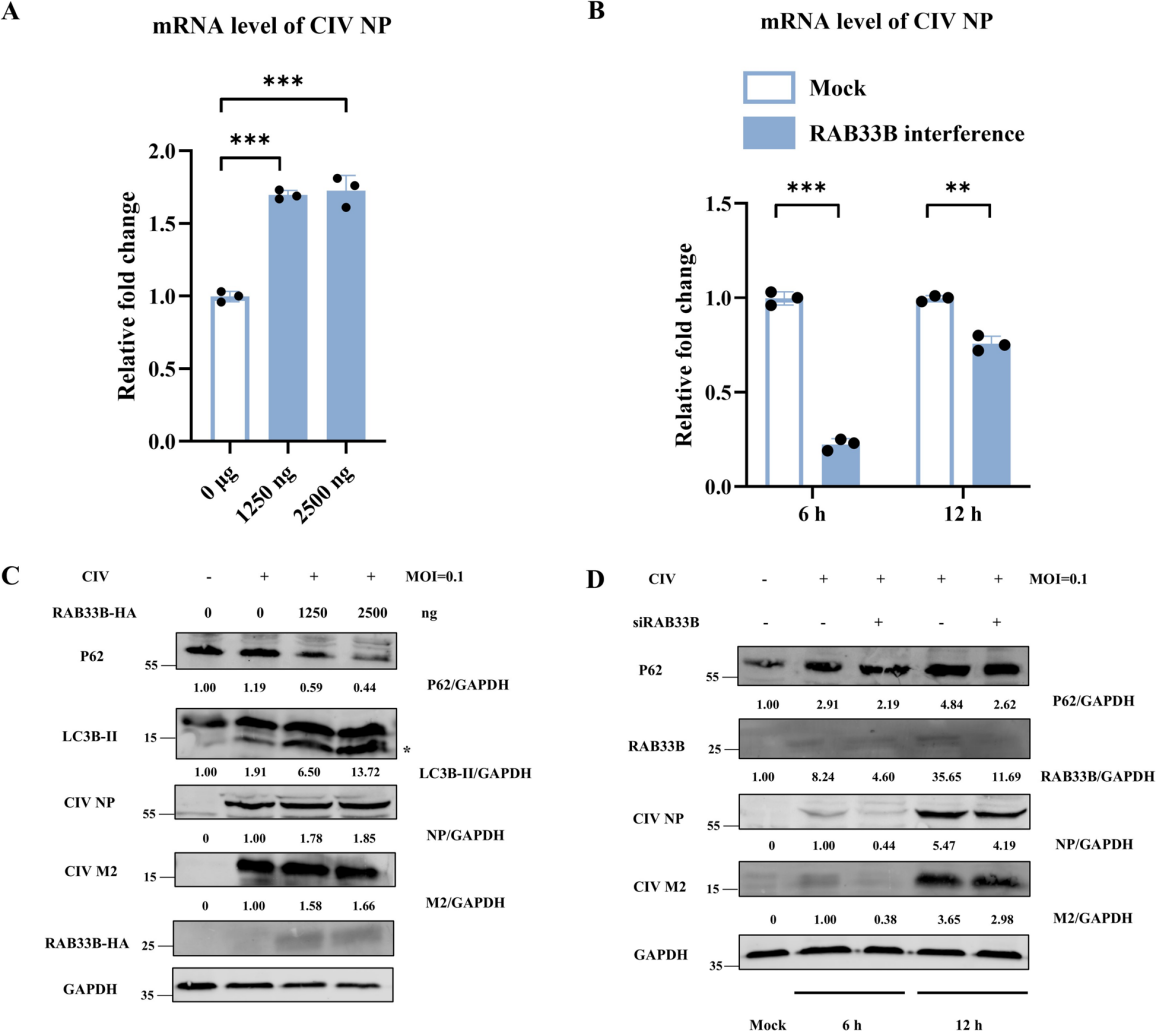
**RAB33B facilitates the replication of CIV. A** HEK293T cells were transfected with different doses of RAB33B plasmid for 24 h and then infected with CIV at MOI = 0.1 for 24 h. **B** HEK293T cells were transfected with siRNA of RAB33B for 24 h and then infected with CIV at MOI = 0.1 for 6 h and 12 h, respectively. **C** HEK293T cells were transfected with different doses of RAB33B plasmid for 24 h and then infected with CIV at MOI = 0.1 for 24 h. Cell lysates were analysed by western blotting. * represents the indicated protein. **D** HEK293T cells were transfected with siRNA of RAB33B for 24 h and then infected with CIV at MOI = 0.1 for 6 h and 12 h, respectively. Cell lysates were analysed by western blotting.

Previous data have shown that CIV M2 increases *RAB33B* gene expression. As RAB33B protein has been proven to promote autophagy [25, 26], we hypothesise that it may enhance autophagy to facilitate CIV replication. However, the effects of RAB33B on human cell autophagy remain largely unknown, as most existing studies have been conducted in mouse cells. To investigate this, we transfected HEK293T cells with a wild-type human RAB33B plasmid, which was found to promote complete autophagy (Additional file 2B). We also observed co-localisation between RAB33B and LC3 through confocal microscopy (Additional file 2A). These data suggested that wild-type human RAB33B positively influences autophagy. To assess the effect of RAB33B on CIV replication, we transfected HEK293T cells with either RAB33B plasmids or RAB33B-targeting siRNA (RNA interference efficacy validated in Additional file 2C). Following CIV infection, we measured replication using qPCR and western blotting. Our results indicated that RAB33B overexpression significantly increased CIV replication in a dose-dependent manner (Figures 3A and C). Conversely, RAB33B silencing inhibited CIV replication, especially early in infection (Figures 3B and D). Additionally, RAB33B knockdown also impaired the replication of H1N1 and H5N1 IAV (Additional file 3). These findings indicate that RAB33B promotes autophagy and CIV replication in host cells and may similarly influence other influenza subtypes.

### RAB33B interacts with CIV M2 and LC3 on vesicles

**Figure 4:**
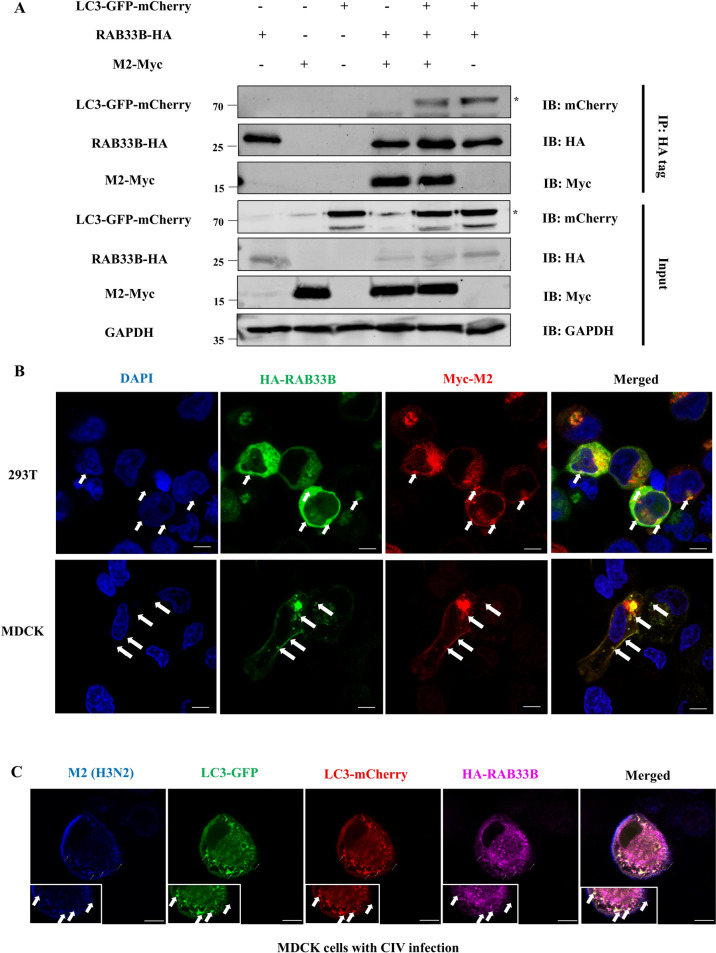
**RAB33B interacts with CIV M2 and LC3 on vesicles. A** HEK293T cells were co-transfected with RAB33B-HA, M2-Myc, and LC3-GFP-mCherry plasmids for 24 h. Cell lysates were subjected to co-immunoprecipitation and western blotting. **B** HEK293T cells were co-transfected with RAB33B-HA and M2-Myc plasmids and analysed for the co-localisation of CIV M2 and LC3. Scale bar: 6 μm. **C** HEK293T cells were co-transfected with RAB33B-HA and LC3-GFP-mCherry plasmids for 24 h and infected with CIV at MOI = 0.1 for 24 h. Cells were analysed for the co-localisation of CIV M2, RAB33B and LC3. Scale bar: 6 μm. The white arrow indicates the co-localisation.

Previous studies show that IAV M2 contains an LC3-interacting region (LIR) and interacts with LC3, similar to the observed co-localisation of RAB33B with LC3 [25]. These findings suggested the potential for interaction among RAB33B, CIV M2, and LC3. To investigate this, HEK293T cells were co-transfected with RAB33B-HA, M2-Myc, and LC3-GFP-mCherry plasmids to confirm this. Co-immunoprecipitation verified the interaction between RAB33B, M2, and LC3 (Figure 4A). Further analysis revealed that overexpressed RAB33B and M2 co-localised and formed aggregates resembling vesicles in HEK293T and MDCK cells. Notably, some of these co-localised vesicles appeared on the inner side of the M2-positive membrane (Figure 4B).

To confirm the subcellular localisation of these interactions during IAV infection, MDCK cells transfected with LC3-GFP-mCherry were infected with different IAV subtypes (H1N1, H3N2, H5N1). The confocal microscopy observation of MDCK cells showed RAB33B co-localising with M2 in the cytoplasm and inner side of the M2-positive membrane (also the LC3-positive membrane) by vesicles. This outcome presented a similar pattern to HEK293T cells overexpressing RAB33B and M2 (Figure 4C and Additional file 4). To determine which M2 protein domain was crucial for the above interaction, we constructed truncated CIV M2 plasmids divided into N-terminal, transmembrane, and C-terminal domains.

HEK293T cells co-transfected with RAB33B-HA, and the truncated M2 plasmids demonstrated that only the C-terminal domain of M2 co-localised with RAB33B (Additional file 5). These findings suggest that RAB33B interacts with CIV M2 and LC3 via the vesicular structures in a manner dependent on the C-terminal domain of M2.

### RAB33B transports CIV M2 protein to the plasma membrane by autophagic-like vesicles

**Figure 5:**
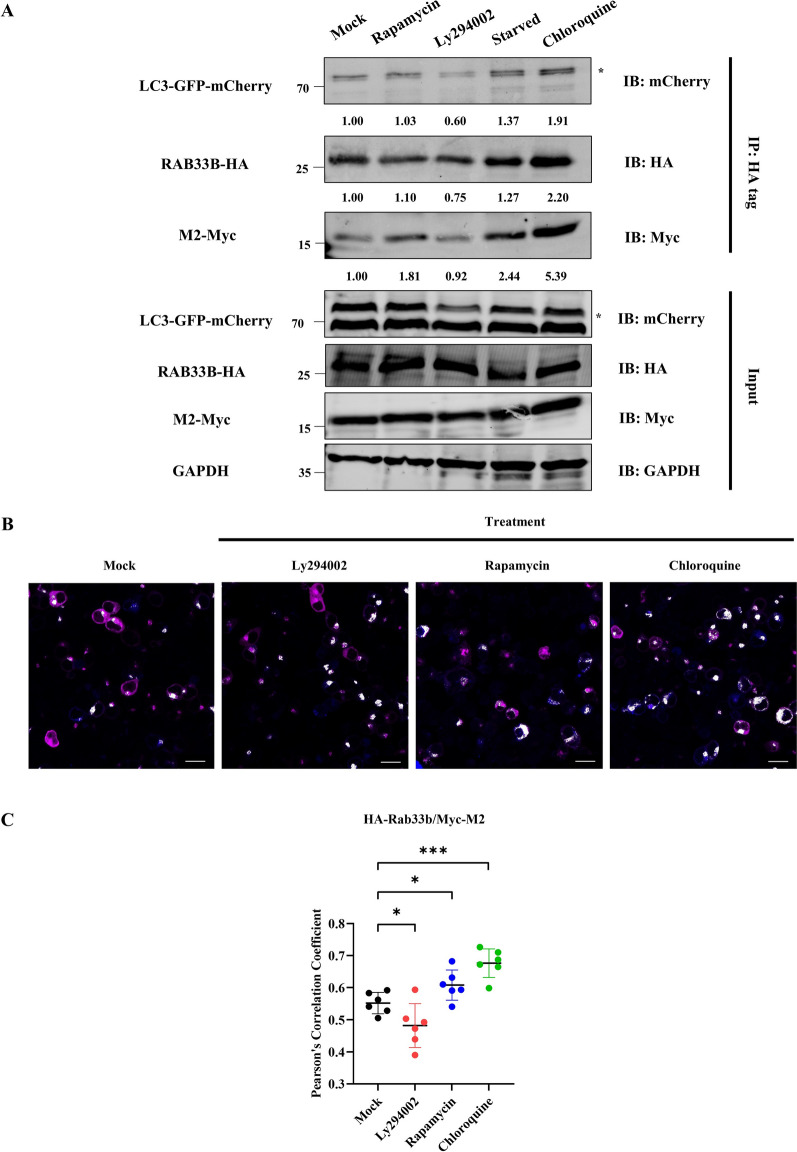
**The interaction of CIV M2, RAB33B and LC3 is regulated by the autophagy process. A** HEK293T cells were co-transfected with RAB33B-HA, M2-Myc, and LC3-GFP-mCherry plasmids and then treated with rapamycin, LY294002, starvation treatment and chloroquine, respectively. Cell lysates were subjected to co-immunoprecipitation and western blotting. **B** HEK293T cells were co-transfected with RAB33B-HA and M2-Myc plasmids and then treated with rapamycin, LY294002 and chloroquine, respectively. The co-localisation between CIV M2 and RAB33B was analysed by Leica LAS X software. Scale bar: 20 μm. The white dots indicated the co-localisation between CIV M2 and RAB33B. **C** The Pearson’s correlation coefficient was analysed from six different fields of vision for **B**, respectively.

Previous data showed that RAB33B interacts with CIV M2 and LC3 via vesicle form. RAB GTPase proteins are known to be transporters for IAV components [23], and other studies suggest that RAB33B participates in exocyst-based post-Golgi transportation [27]. In our live-cell imaging analysis, we observed that CIV M2 could be transported to the plasma membrane through vesicle form. The size of these vesicles was similar to that of autophagosomes (Additional file 15 and Additional file 16). Based on these findings and existing literature, we hypothesised that RAB33B would execute a membrane transport function for CIV M2 and might be linked to autophagy progression. To explore this, HEK293T cells co-transfected with RAB33B-HA, M2-Myc, and LC3-GFP-mCherry plasmids were treated with drugs to simulate early-autophagy inhibition, activation, and late inhibition. The co-immunoprecipitation result showed that LY294002 reduced RAB33B, M2, and LC3 interactions in early autophagy, whereas rapamycin and starvation enhanced them. Notably, the most robust interactions were observed following chloroquine treatment, which blocks late autophagy by altering the pH of the autolysosome (Figure 5A). Confocal co-localisation analysis confirmed that chloroquine treatment produced the highest level of RAB33B and CIV M2 co-localisation (Figures 5B and C). These results suggest that RAB33B, M2, and LC3 interactions are autophagy-progression dependent and play a role when late-stage autolysosome formation is impaired.

**Figure 6:**
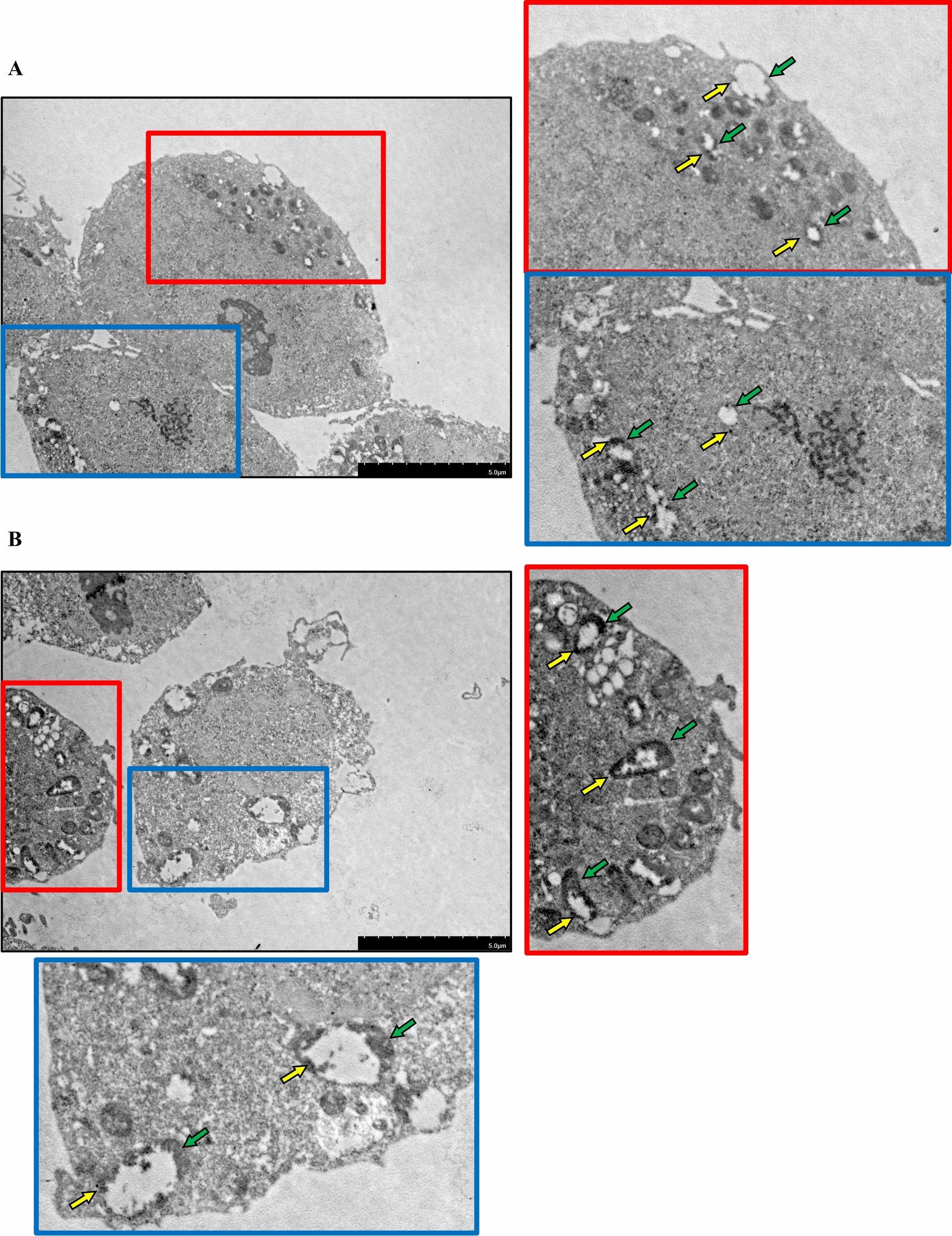
**Immunogold transmission electron microscopy observation for the CIV-infected HEK293T cells. A** represents the mock group. **B** represents the cells transfected with RAB33B plasmid. Scale bar: 5 μm. The yellow arrow indicated the CIV M2, and the green arrow indicated the autophagic-like vesicles.

However, these findings did not fully substantiate that RAB33B transported M2 protein. To investigate this, we conducted immunogold transmission electron microscopy to observe the CIV-infected HEK293T cells with and without RAB33B overexpression. Our results showed that RAB33B overexpression increases the number of vesicles located near the plasma membrane’s inner side. M2 protein was observed on both the autophagic-like vesicles’ outer and intracavitary membranes (Figure 6). The above phenomenon indicates that RAB33B may transport M2 to the membrane via these special vesicles by a possible autophagy-related process. Further 3D reconstruction using confocal microscopy in CIV-infected MDCK cells overexpressing RAB33B showed punctate RAB33B localisation on the inner plasma membrane, with CIV M2 fluorescence co-localising at these vesicle-like sites (Additional file 6, Additional file 12, Additional file 13).

Collectively, these findings support a model in which RAB33B transports CIV M2 intracellularly via the autophagic-like vesicles. In brief, RAB33B transports CIV M2 to the plasma membrane through an autophagy-based process.

### The CIV M2 protein trafficking involves the RAB33B effector protein ATG16L1

**Figure 7:**
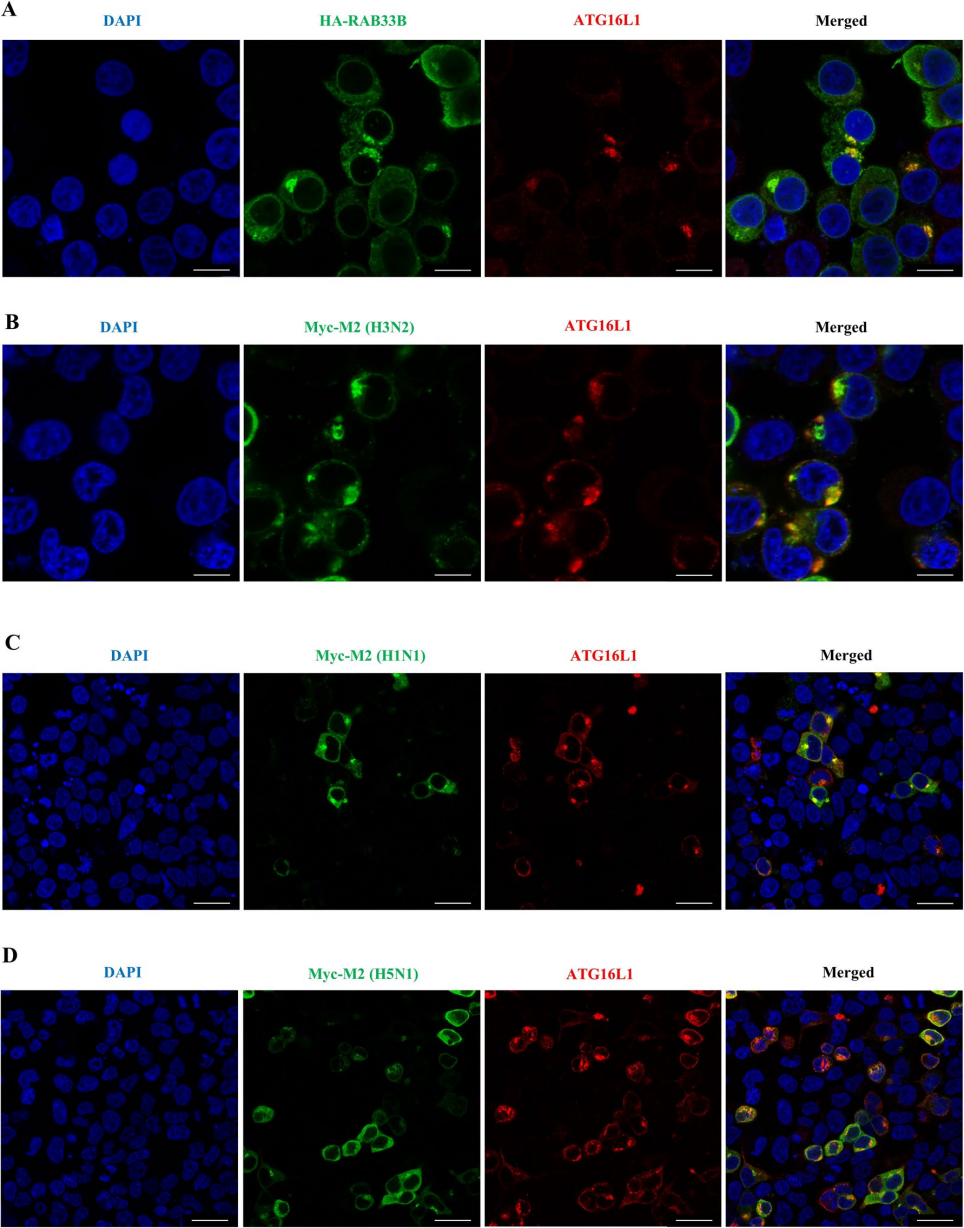
**The redistribution of ATG16L1 induced by RAB33B and M2, respectively. A** HEK293T cells were transfected with RAB33B-HA. Scale bar: 10 μm. **B** HEK293T cells were transfected with H3N2 CIV M2 plasmid. Scale bar: 10 μm. **C** HEK293T cells were transfected with H1N1 IAV M2 plasmid. Scale bar: 20 μm. **D** HEK293T cells were transfected with the H5N1 subtype of IAV M2 plasmid. Scale bar: 20 μm.

Early data revealed that CIV M2 not only promotes autophagy but also regulates ATG16L1 protein expression (Figure 1 and Additional files 14A and B). ATG16L1, an effector of RAB33B, is recruited to the phagophores by RAB33B [26]. Therefore, we hypothesised that ATG16L1 might play a role in RAB33B-mediated CIV M2 protein transport. To test this, we compared ATG16L1’s subcellular localisation in HEK293T cells transfected with RAB33B-HA or M2-Myc. The results showed that RAB33B overexpression led to ATG16L1 aggregation near the nucleus (Figure 7A). Conversely, CIV M2 overexpression caused ATG16L1 to aggregate in the cytoplasm and near the plasma membrane, mimicking LC3 redistribution by IAV M2 (Figure 7B). This phenomenon was consistent across H1N1 and H5N1 M2-transfected cells (Figures 7C and D). We examined RAB33 B’s role in M2-induced ATG16L1 redistribution by observing increased RAB33B and ATG16L1 co-localisation in autophagic-like vesicles in CIV-infected MDCK cells. Moreover, we found that autophagic-like vesicles near the plasma membrane increased over time post-infection (Additional file 7). These results suggested that ATG16L1 was involved in RAB33B-mediated IAV M2 protein trafficking.

### GTPase-activating protein TBC1D25 of RAB33B regulates the CIV replication

**Figure 8:**
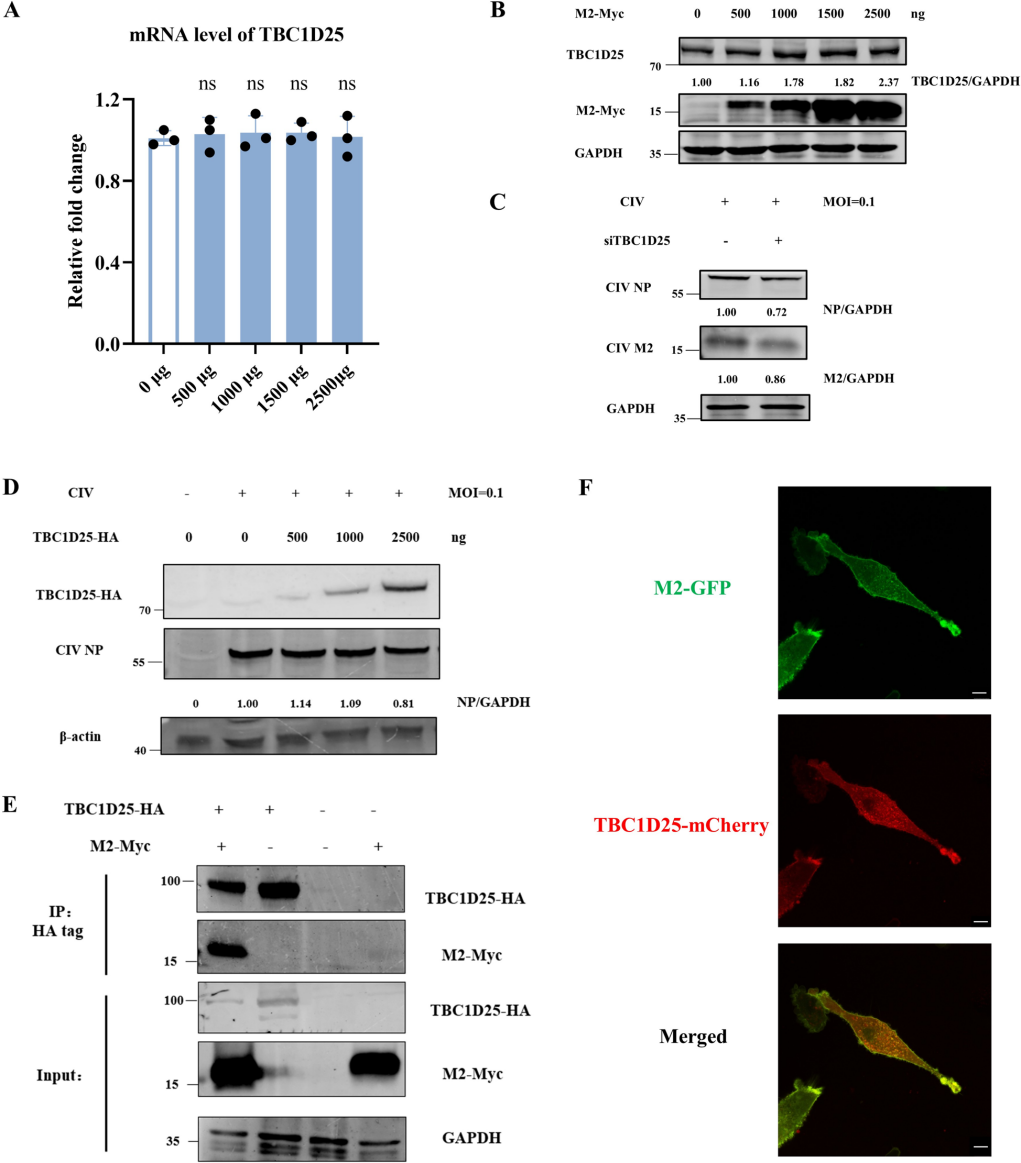
**GTPase-activating protein TBC1D25 of RAB33B regulates the CIV replication. A** HEK293T cells were transfected with CIV M2 plasmid for 24 h and were analysed by qPCR. **B** HEK293T cells were transfected with CIV M2 plasmid for 24 h. Cell lysates were analysed by western blotting. **C** HEK293T cells were transfected with siRNA of TBC1D25 for 24 h and then infected with CIV at MOI = 0.1 for 24 h. Cell lysates were analysed by western blotting. **D** HEK293T cells were transfected with different doses of TBC1D25 plasmid for 24 h and then infected with CIV at MOI = 0.1 for 24 h. Cell lysates were analysed by western blotting. **E** HEK293T cells were co-transfected with TBC1D25-HA and M2-Myc plasmids for 24 h. Cell lysates were subjected to co-immunoprecipitation and western blotting. **F** MDCK-TetOn-M2 cells were transfected with TBC1D25-mCherry and activated to express CIV M2-GFP by doxycycline for 48 h. Cells were analysed for the co-localisation of CIV M2 and TBC1D25. Scale bar: 10 μm.

Having established the role of RAB33B in CIV M2 transport and the involvement of ATG16L1, we next examined whether RAB33B’s GTPase-activating protein, TBC1D25, also contributes to CIV replication. To investigate, we overexpressed CIV M2 in HEK293T cells and *TBC1D25* expression was assessed by qPCR and western blotting. While transcriptional levels of *TBC1D25* remained unchanged with varying CIV M2 overexpression levels (Figure 8A), protein expression increased in a dose-dependent manner with CIV M2 plasmid transfection (Figure 8B). To assess the functional relevance of *TBC1D2*, we silenced its expression in HEK293T cells and then infected the cells with CIV. Viral replication was significantly reduced (Figure 8C). Interestingly, low-dose overexpression of TBC1D25 promoted viral replication, whereas high-dose overexpression did not (Figure 8D), suggesting a dose-sensitive regulatory effect. In the HEK293FT/MDCK-TetOn-M2 cell lines, the increased TBC1D25 was also detected during the induction of M2-GFP (Additional files 14A and B).

**Figure 9:**
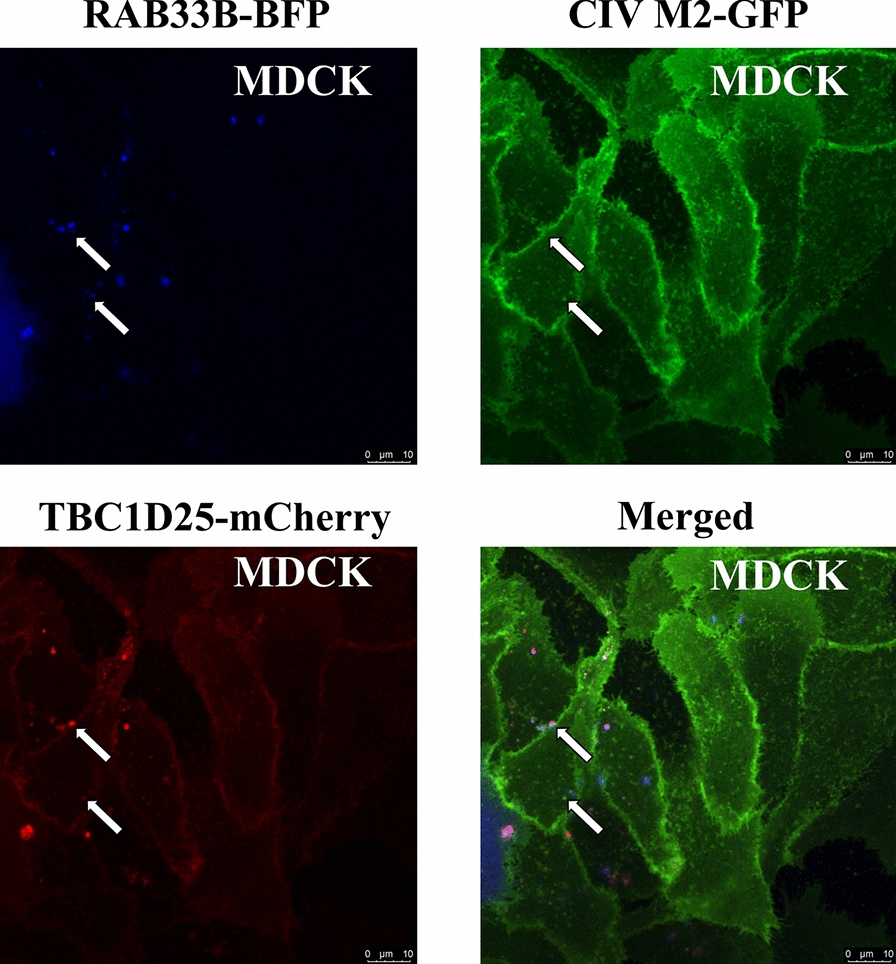
**Interaction among RAB33B, TBC1D25 and CIV M2.** MDCK-TetOn-M2 cells were transfected with RAB33B-BFP and TBC1D25-mCherry and activated to express CIV M2-GFP by doxycycline for 48 h. Cells without fixation were directly observed by a confocal laser scanning microscope. The white arrow indicates the co-localisation. Scale bar: 10 μm.

Co-immunoprecipitation and co-localisation studies confirmed that TBC1D25 interacts with CIV M2 (Figures 8E and F). To examine TBC1D25’s role in CIV M2 membrane trafficking, we conducted live-cell imaging on MDCK-TetOn-M2 cells transfected with RAB33B-BFP and TBC1D25-mCherry. The result revealed co-localisation of RAB33B-BFP, CIV M2-GFP, and TBC1D25-mCherry in the cytoplasm and on the M2-positive plasma membrane (Figure 9). These findings indicated that CIV M2 regulates TBC1D25 protein levels, and TBC1D25 enhances CIV replication through its GAP function for RAB33B.

## Discussion

This study investigated the role of autophagy in IAV infection, using avian-origin CIV as a representative strain. To elucidate how CIV induces autophagy, we first explored changes in autophagy-related protein expression following infection. Our findings verified that CIV infection benefits from autophagy, initiating the process and redistributing the LC3 protein, which is consistent with previous studies [8, 14, 24, 28, 29]. We identified CIV M2 as responsible for CIV infection-induced up-regulated expression of ATG16L1, P62, and LC3, the essential proteins in autophagosome formation and autophagic degradation. Notably, prior studies have shown that silencing ATG16L1 weakens IAV-induced autophagosome formation and normal IAV budding [8]. Our data suggest that CIV M2 positively regulates autophagosome formation through ATG16L1, advancing our understanding of the ATG16L1-IAV relationship. However, other studies indicate that the WD repeat-containing C-terminal domain of ATG16L1 may inhibit IAV infection via non-canonical autophagy [30, 31]. These seemingly contradictory findings imply that ATG16L1 may have multiple roles in IAV-induced autophagy. This complexity highlights the need for further investigation into the context-dependent functions of ATG16L1 during influenza virus infection.

Furthermore, our transcriptomic analysis of two datasets revealed that RAB GTPase proteins contribute to IAV M2 overexpression and CIV infection [32]. This protein family is known for its involvement in multiple autophagy processes [19, 33]. Among these, we found significant up-regulation of the RAB33B gene in datasets, which has been implicated in pro-autophagosome formation [25, 26]. Further experiments confirmed that IAV M2 could crucially up-regulate RAB33B expression in a time- and dose-dependent manner. Prior studies have reported that RAB33B facilitates hepatitis B virus replication [34, 35], suggesting a potential role in viral lifecycle regulation. To assess its function in IAV infection, we conducted overexpression and silencing experiments, demonstrating that increased RAB33B expression promotes IAV replication through autophagy stimulation. However, the precise mechanism by which RAB33B-mediated autophagy facilitates IAV replication remains unclear.

Given that RAB33B has been shown to co-localise with LC3 [25] and that IAV M2 is capable of interacting with LC3 [8, 14], we hypothesised a potential interaction between these proteins. Co-immunoprecipitation experiments and confocal microscopy confirmed that IAV M2 and RAB33B co-exist with LC3, including some interactions occurring on the plasma membrane’s inner side. These findings are consistent with previous studies indicating that IAV M2 can redistribute the localisation of LC3 to produce stable IAV virions [16]. Our findings in this study may explain how RAB33B assists IAV M2 in modulating the redistribution of LC3 to maintain IAV virion stability by enhancing autophagy. We also found that the co-localisation of IAV M2 and RAB33B is dependent on IAV M2’s C-terminal. Proteins with LIR motifs are known to interact with each other [36, 37]. Although both RAB33B and IAV M2 have the LIR motif, whether their interaction relies on IAV M2’s LIR motif remains uncertain and will be explored in future studies.

RAB GTPase proteins are beneficial to the membrane trafficking of IAV proteins, with prior research underscoring their regulatory importance at all stages of IAV infection [23]. For example, RAB5 and RAB7 mediate IAV virus entry, fusion, and uncoating, corresponding to early and late endosomes [21], while RAB17 and RAB23 transport IAV HA and NA proteins to the plasma membrane [39]. RAB33B is a protein primarily associated with Golgi-to-endoplasmic reticulum transport [40]. Recent studies have shown that it interacts with the exocyst complex, mediating cell-localised secretion involved in focal adhesion turnover and cell migration [27]. Building on these findings, we hypothesised that RAB33B also functions as a transporter for IAV infection by autophagy-related vesicles. To investigate this, we further explored RAB33B’s role in IAV M2 membrane trafficking through experiments. We found that early autophagy stimulation and late autophagy inhibition enhanced the interaction of IAV M2, RAB33B, and LC3. Our previous data indicated that IAV M2 induced autophagy-related vesicle (containing LC3) accumulation during the IAV infection. Together, these findings suggest the interaction is related to inhibited autophagy in the late stage, correlating with IAV M2-induced autophagosome and autolysosome accumulation.

Results of immunogold electron microscopy further revealed that RAB33B overexpression increased the number of autophagic-like vesicles near the plasma membrane and the aggregation of IAV M2 on their outer membrane. Moreover, fluorescence observation confirmed that RAB33B and IAV M2 interact via autophagic-like vesicles on the plasma membrane’s inner side. However, the precise molecular regulation by which RAB33B-mediated autophagy-related vesicles transport IAV M2 to the plasma membrane remains unclear. Studies have indicated that the exocyst complex is involved in autophagy [41, 42]. In addition, the exocyst-myosin V pathway, RAB11-mediated trafficking of respiratory syncytial virus proteins [43]. Given the limited reports and knowledge, our findings raise the possibility that an unidentified process for IAV M2 trafficking involves an RAB33B-mediated exocyst pathway linked to autophagy.

RAB GTPase proteins alternate between GTP- and GDP-bound states, regulated by guanine nucleotide exchange factors (GEFs) and GTPase-activating proteins (GAPs), respectively. The function of RAB33B is mediated through interactions with effectors. ATG16L1 and TBC1D25 have been identified as its effector and GAP, respectively [44, 45]. Subsequent experiments revealed that with various subtypes of IAV infections, ATG16L co-localised with RAB33B in autophagic-like vesicles, and ATG16L1 was redistributed alongside IAV M2. These results suggest ATG16L1 is involved in the interaction between RAB33B and IAV M2, although the precise mechanism remains to be determined.

Additionally, IAV M2 increased TBC1D25 protein expression without affecting its transcription level. This outcome indicates that IAV M2 modulates TBC1D25 at the post-transcriptional level. Furthermore, TBC1D25 deficiency impaired IAV replication. Previous studies have shown that TBC1D25 delays autophagosomal maturation and may localise to the outer phagophore to evade degradation, owing to its lack of a complete LIR and necessary oligomerisation [46]. We confirmed the interaction between IAV M2 and TBC1D25, and live-cell imaging showed that RAB33B, IAV M2, and TBC1D25 co-localise at the inner plasma membrane side in MDCK-TetOn-M2 cells transfected with RAB33B-BFP and TBC1D25-mCherry. Notably, TBC1D25 overexpression exerted opposite effects on IAV replication depending on the dose. This phenomenon might stem from TBC1D25’s GAP function, which converts GTP-RAB33B to GDP-RAB33B, thereby inactivating RAB33B, inhibiting RAB33B-mediated autophagy. Thus, the appropriate expression of TBC1D25 assisted RAB33B in mediating the membrane trafficking of IAV M2 through its GAP function.

**Figure 10:**
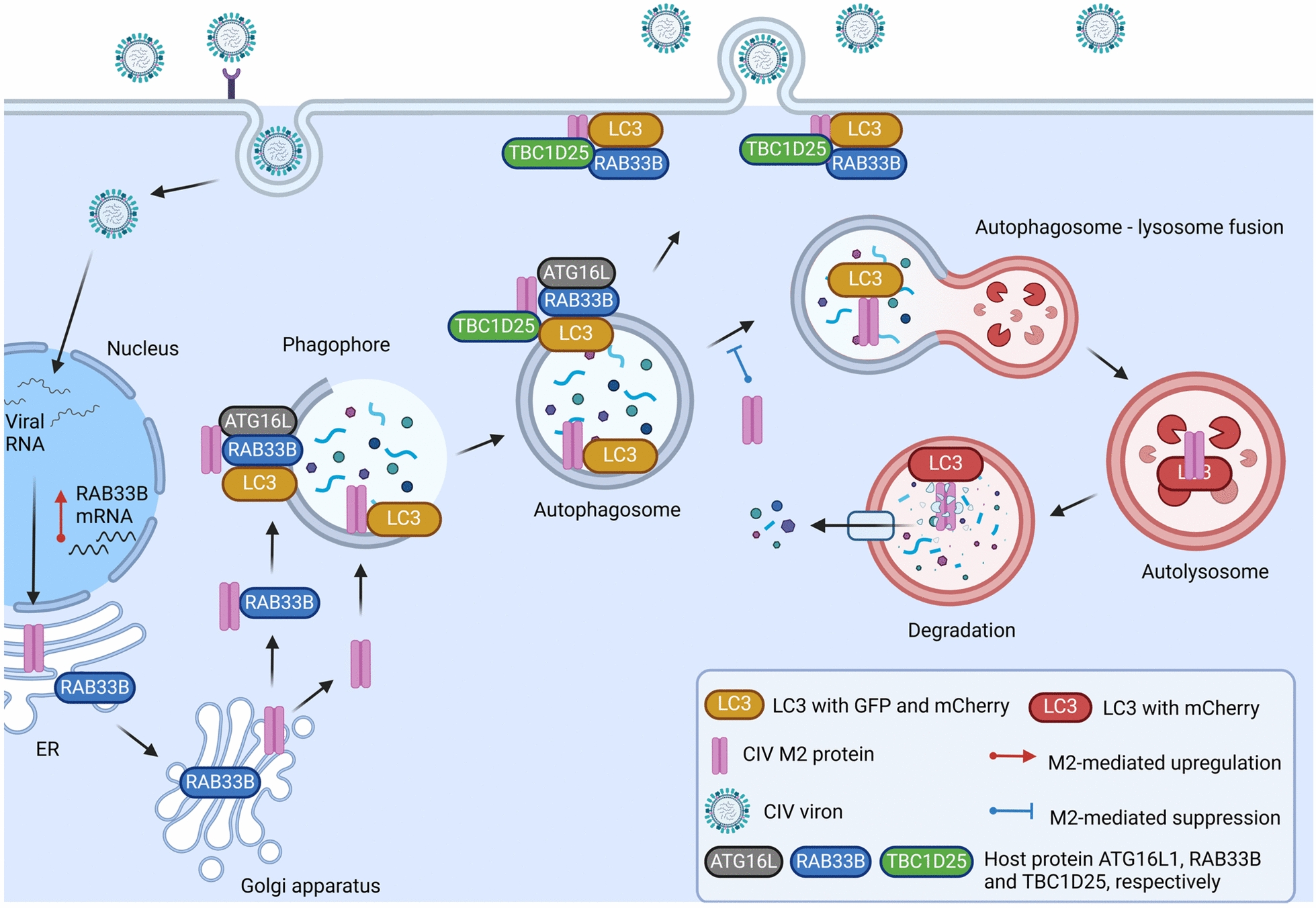
**Schematic diagram of proposed mechanisms underlying RAB33B-mediated Influenza A virus M2 trafficking**.

In summary, this study emphasises IAV’s unique manipulation of host autophagy, with a specific focus on the role of RAB33B in promoting IAV replication through the regulation of autophagy for IAV M2 protein membrane trafficking (Figure 10). Our findings contribute to and advance the understanding of how IAV utilises host autophagy to facilitate its replication, thus suggesting potential targets for developing future antiviral interventions.

## Supplementary Information

**Additional file 1.** **Next-generation sequencing analysis of CIV M2 overexpression in HEK293T cells.** **A **Clustering analysis heatmap of DEGs. **B **Volcano plot visualisation of DEGs. **C **Analysis of GO enrichment in molecular function, cellular component, and biological process.

**Additional file 2**. **RAB33B regulates autophagy in HEK293T cells. A** HEK293T cells were co-transfected with RAB33B-HA and LC3-GFP-mCherry plasmids for 24 h and analysed for the co-localisation of CIV and LC3. Scale bar: 10 μm. **B **HEK293T cells were transfected with different doses of RAB33B-HA plasmids, and cell lysates were analysed using western blotting. **C **HEK293T cells were transfected with different siRNA of RAB33B for 24 h. The relative mRNA expression levels of RAB33B were analysed by qPCR.

**Additional file 3.** **The deficiency of RAB33B impairs H1N1 and H5N1 IAV replication in HEK293T. A **HEK293T cells were transfected with siRNA of RAB33B for 24 h and then infected with H1N1 IAV at MOI = 5 for 12 h. Cell lysates were subjected to western blotting. **B **HEK293T cells were transfected with siRNA of RAB33B for 24 h and then infected with H5N1 IAV at MOI = 5 for 12 h. Cell lysates were subjected to western blotting.

**Additional file 4.** **Interaction among RAB33B, LC3 and H1N1/H5N1 M2 in infected MDCK cells.** MDCK cells were transfected with RAB33B-HA and LC3-GFP-mCherry plasmids for 24 h and infected with the indicated subtype of IAV at MOI = 0.1 for 24 h. Cells were analysed for the co-localisation of M2, RAB33B and LC3. Scale bar: 10 μm. The white arrow indicates co-localisation.

**Additional file 5.** **Truncated C-terminal M2 co-localised with RAB33B. A** Three domains of CIV M2 protein. **B **HEK293T cells were transfected with different truncated M2-Myc and RAB33B-HA plasmids. Cells were analysed for the co-localisation of truncated M2 and RAB33B. Scale bar: 10 μm.

**Additional file 6**. **RAB33B helps CIV M2 protein trafficking to the plasma membrane.** HEK293T cells were transfected with RAB33B-HA plasmid for 24 h and then infected with CIV at MOI = 0.1 for 24 h. Cells were analysed for the co-localisation of truncated M2 and RAB33B. **A **3D reconstruction of z-stack, field 1. **B **3D reconstruction of z-stack, field 2.

**Additional file 7**. **CIV infection activates and redistributes RAB33B and ATG16L1 protein.** MDCK cells were infected with CIV at MOI = 0.1 for 6 h and 12 h, respectively. Cells were analysed for the co-localisation of RAB33B and ATG16L1. Scale bar: 20 μm.

**Additional file 8.**  **Plasmids used in this study**.

**Additional file 9.** **Primers used in this study. **Primer sets used for qPCR in this study.

**Additional file 10**. **siRNA sequences used in this study.** Sequence of siRNA used in this study.

**Additional file 11.** **Transcriptome analysis of Mock vs M2 in HEK293T cells.** The differentially expressed genes of overexpression of IAV M2 in HEK293T cells compared to the Mock group.

**Additional file 12.** **The video of the z-stack field of CIV-infected MDCK transfected with RAB33B-HA plasmid.** The co-localisation fluorescence showed RAB33B protein forming spots on the plasma membrane’s inner side. M2 protein fluorescence co-localised with these vesicle-like spots. Scale bar: 5 μm.

**Additional file 13**. **RAB33B-positive vesicles are localised on the M2-positive plasma membrane’s inner side.** The co-localisation fluorescence indicated that RAB33B protein-positive vesicles were localised on the M2-positive plasma membrane’s inner side. WGA was used to stain the plasma membrane. The white arrow indicates the fluorescence signal on the plasma membrane’s inner side. The yellow arrow indicates the fluorescence signal around the nucleus. Scale bar: 5 μm.

**Additional file 14**. **The establishment of HEK293FT/MDCK-TetOn-M2 cell line.** **A **HEK293FT-TetOn-M2 cells were activated to express CIV M2-GFP by doxycycline for 48 h. Cell lysates were analysed by western blotting. * represents the indicated protein. **B **MDCK-TetOn-M2 cells were activated to express CIV M2-GFP by doxycycline for 48 h. Cell lysates were analysed by western blotting. * represents the indicated protein. **C **The stable expression of M2-GFP induced by doxycycline was confirmed. Scale bar: 10 μm.

**Additional file 15**. **Live-cell imaging of M2-GFP induced by doxycycline in HEK293FT-TetOn-M2 cell line**. HEK293FT-TetOn-M2 cells were activated to express CIV M2-GFP by doxycycline for 48 h. The M2-GFP signal was captured by live-cell observation. Scale bar: 10 μm.

**Additional file 16**. **Live-cell imaging of M2-GFP induced by doxycycline in MDCK-TetOn-M2 cell line**. MDCK-TetOn-M2 cells were activated to express CIV M2-GFP by doxycycline for 48 h. The M2-GFP signal was captured by live-cell observation. Scale bar: 10 μm.

## Data Availability

All data generated or analyzed during this study are included in this published article and its supplementary information files. If necessary, please contact the corresponding author for more data.
