# Supplementary figures and images for: Host cellular protein RAB33B facilitates influenza viral replication and modulates M2 trafficking by enhancing autophagy
Source: Vet Res. 2025 Jul 1;56:129. doi: 10.1186/s13567-025-01560-6 (PMC12219998; doi:10.1186/s13567-025-01560-6)

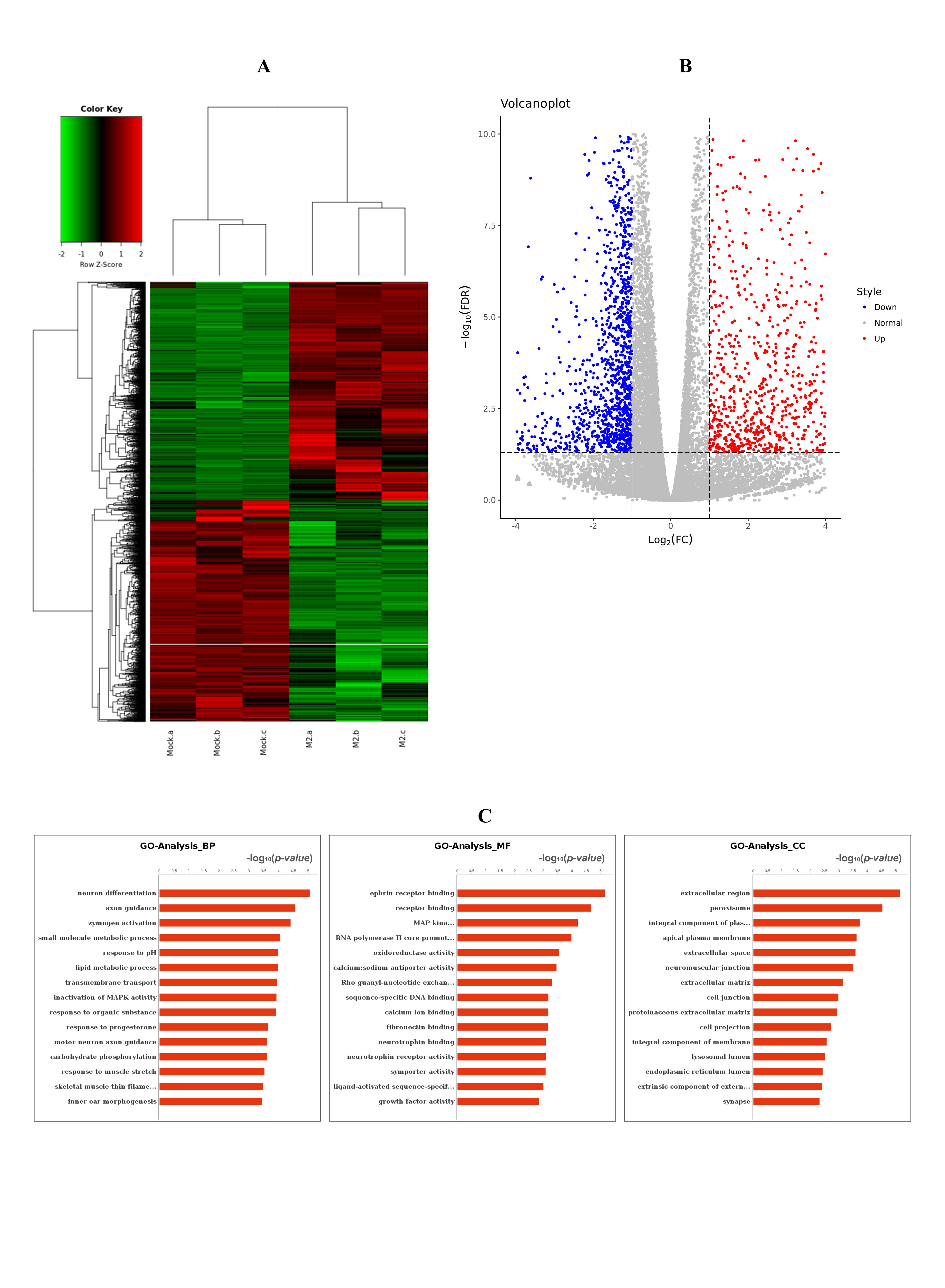

Supplement: Supplementary file 1 — Additional file 1. Next-generation sequencing analysis of CIV M2 overexpression in HEK293T cells. A Clustering analysis heatmap of DEGs. B Volcano plot visualisation of DEGs. C Analysis of GO enrichment in molecular function, cellular component, and biological process. [file 13567_2025_1560_MOESM1_ESM.jpg]

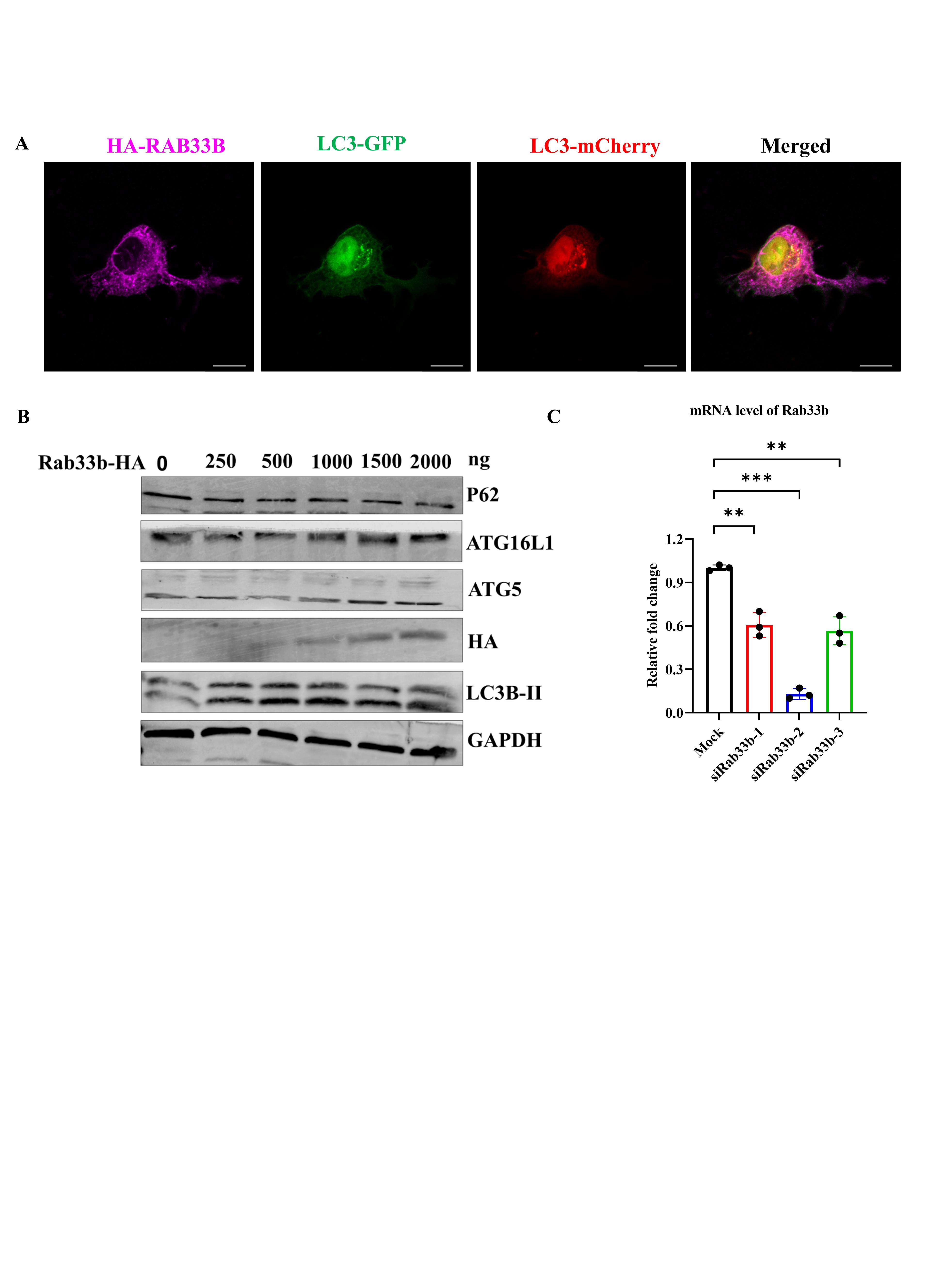

Supplement: Supplementary file 2 — Additional file 2. RAB33B regulates autophagy in HEK293T cells. A HEK293T cells were co-transfected with RAB33B-HA and LC3-GFP-mCherry plasmids for 24 h and analysed for the co-localisation of CIV and LC3. Scale bar: 10 μm. B HEK293T cells were transfected with different doses of RAB33B-HA plasmids, and cell lysates were analysed using western blotting. C HEK293T cells were transfected with different siRNA of RAB33B for 24 h. The relative mRNA expression levels of RAB33B were analysed by qPCR. [file 13567_2025_1560_MOESM2_ESM.jpg]

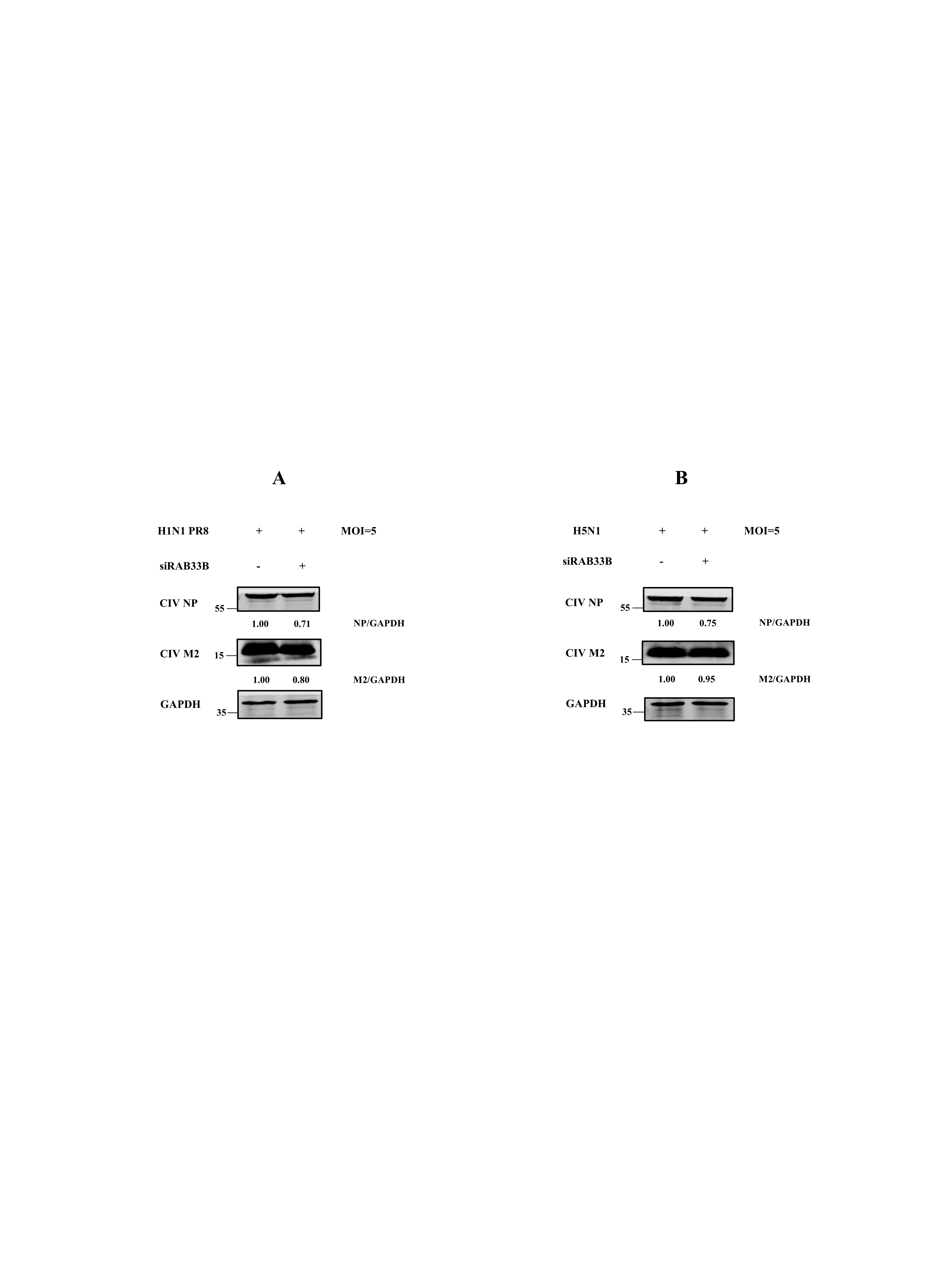

Supplement: Supplementary file 3 — Additional file 3. The deficiency of RAB33B impairs H1N1 and H5N1 IAV replication in HEK293T. A HEK293T cells were transfected with siRNA of RAB33B for 24 h and then infected with H1N1 IAV at MOI = 5 for 12 h. Cell lysates were subjected to western blotting. B HEK293T cells were transfected with siRNA of RAB33B for 24 h and then infected with H5N1 IAV at MOI = 5 for 12 h. Cell lysates were subjected to western blotting. [file 13567_2025_1560_MOESM3_ESM.jpg]

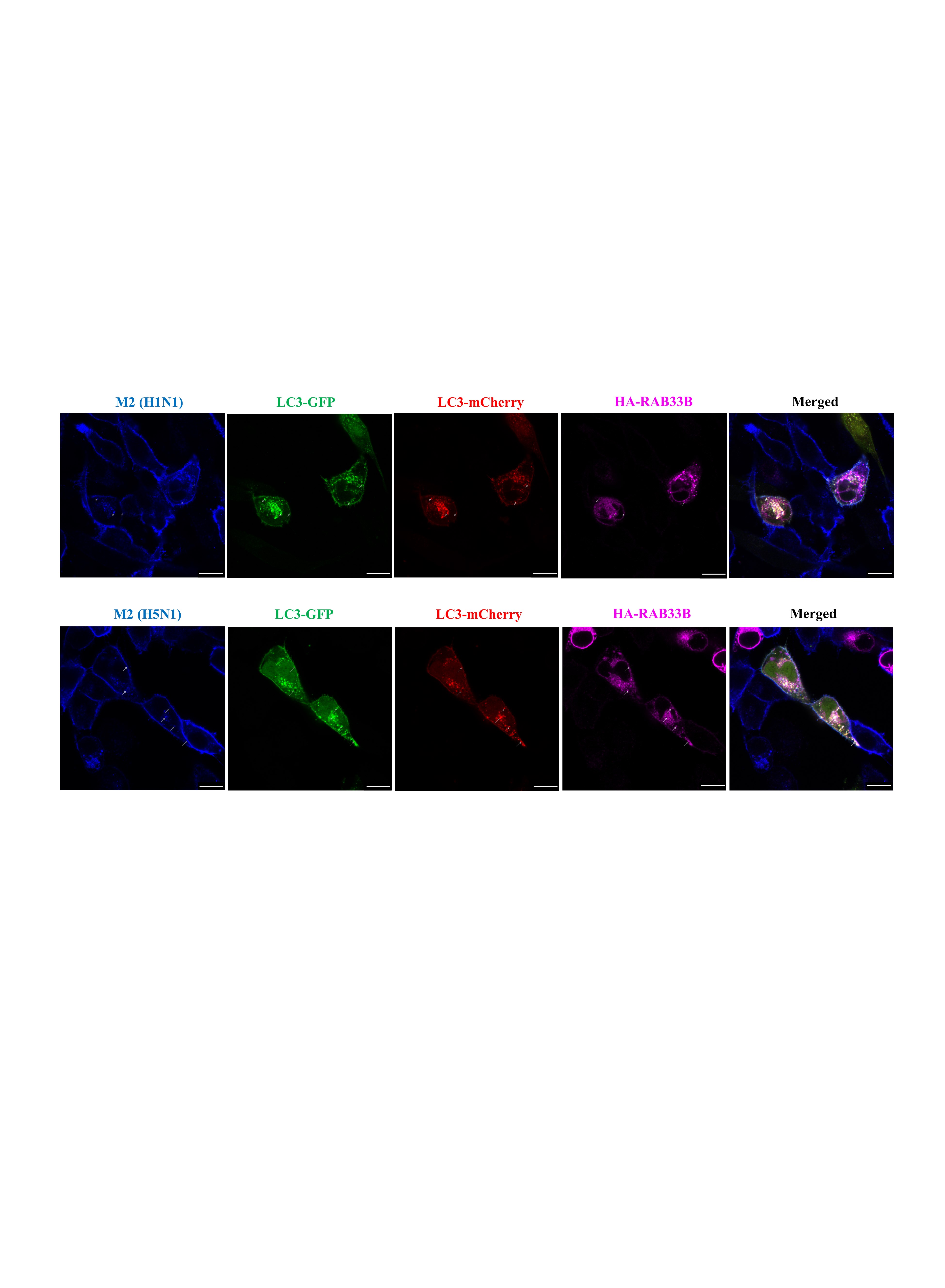

Supplement: Supplementary file 4 — Additional file 4. Interaction among RAB33B, LC3 and H1N1/H5N1 M2 in infected MDCK cells. MDCK cells were transfected with RAB33B-HA and LC3-GFP-mCherry plasmids for 24 h and infected with the indicated subtype of IAV at MOI = 0.1 for 24 h. Cells were analysed for the co-localisation of M2, RAB33B and LC3. Scale bar: 10 μm. The white arrow indicates co-localisation. [file 13567_2025_1560_MOESM4_ESM.jpg]

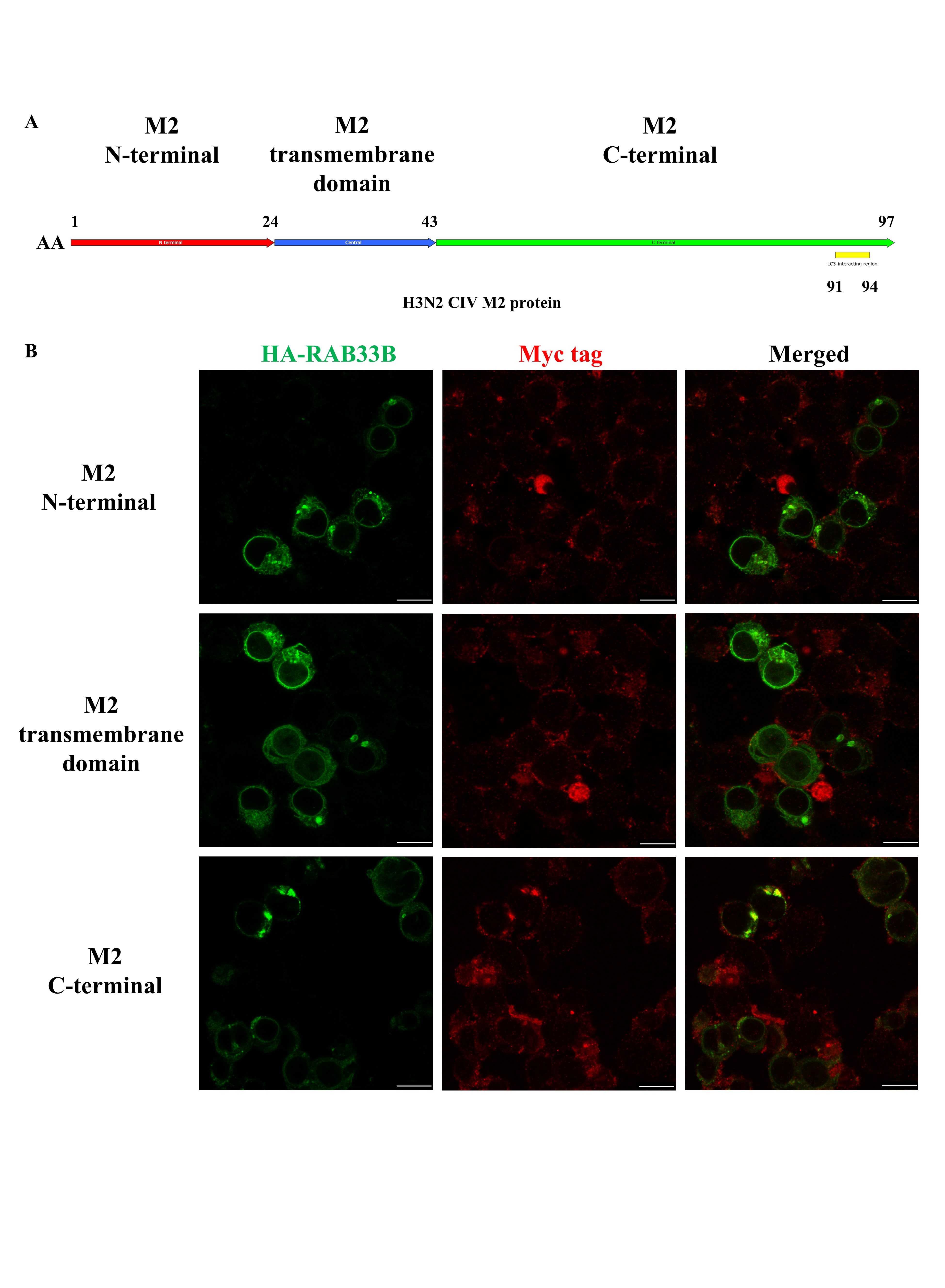

Supplement: Supplementary file 5 — Additional file 5. Truncated C-terminal M2 co-localised with RAB33B. A Three domains of CIV M2 protein. B HEK293T cells were transfected with different truncated M2-Myc and RAB33B-HA plasmids. Cells were analysed for the co-localisation of truncated M2 and RAB33B. Scale bar: 10 μm. [file 13567_2025_1560_MOESM5_ESM.jpg]

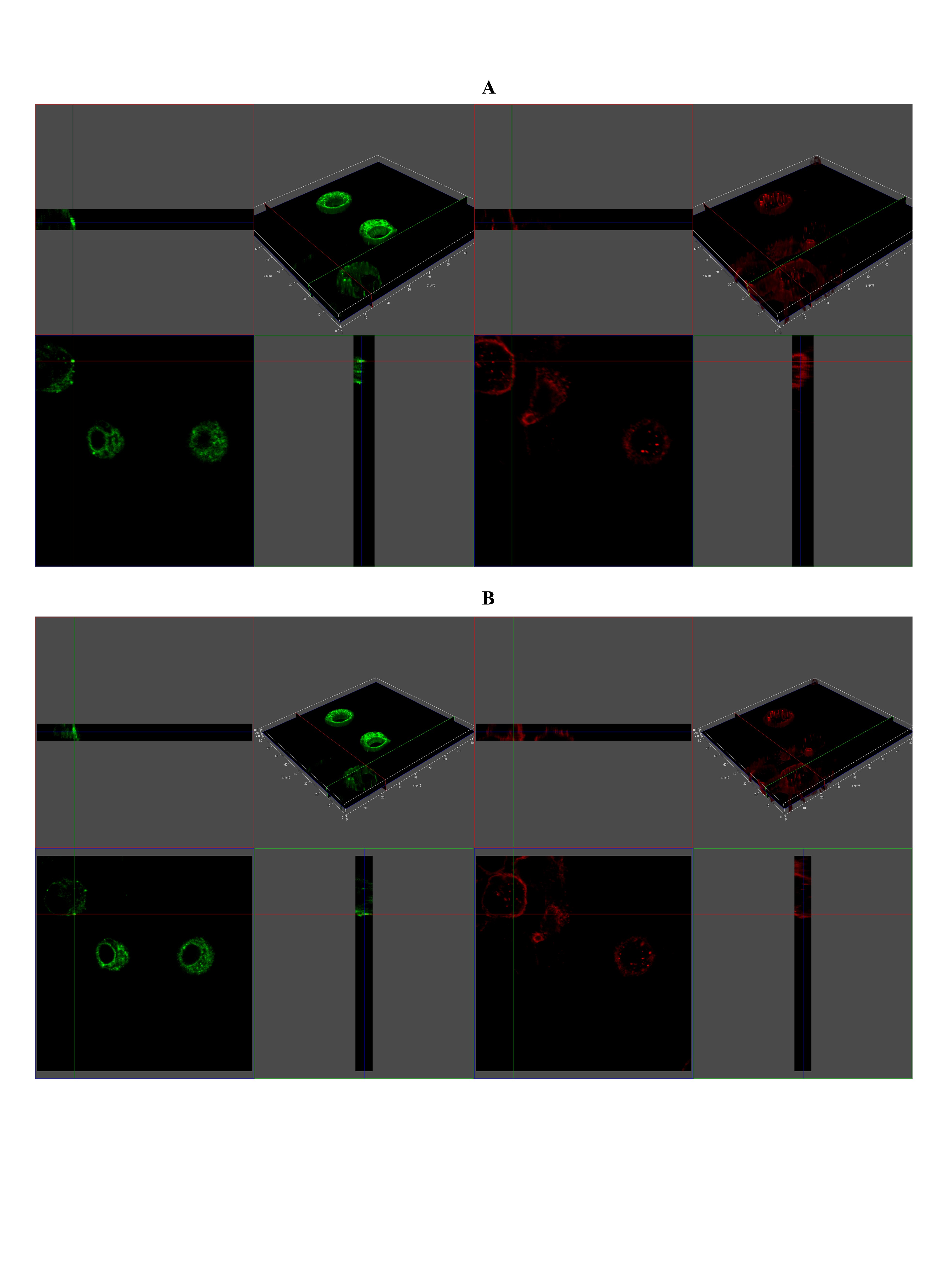

Supplement: Supplementary file 6 — Additional file 6. RAB33B helps CIV M2 protein trafficking to the plasma membrane. HEK293T cells were transfected with RAB33B-HA plasmid for 24 h and then infected with CIV at MOI = 0.1 for 24 h. Cells were analysed for the co-localisation of truncated M2 and RAB33B. A 3D reconstruction of z-stack, field 1. B 3D reconstruction of z-stack, field 2. [file 13567_2025_1560_MOESM6_ESM.jpg]

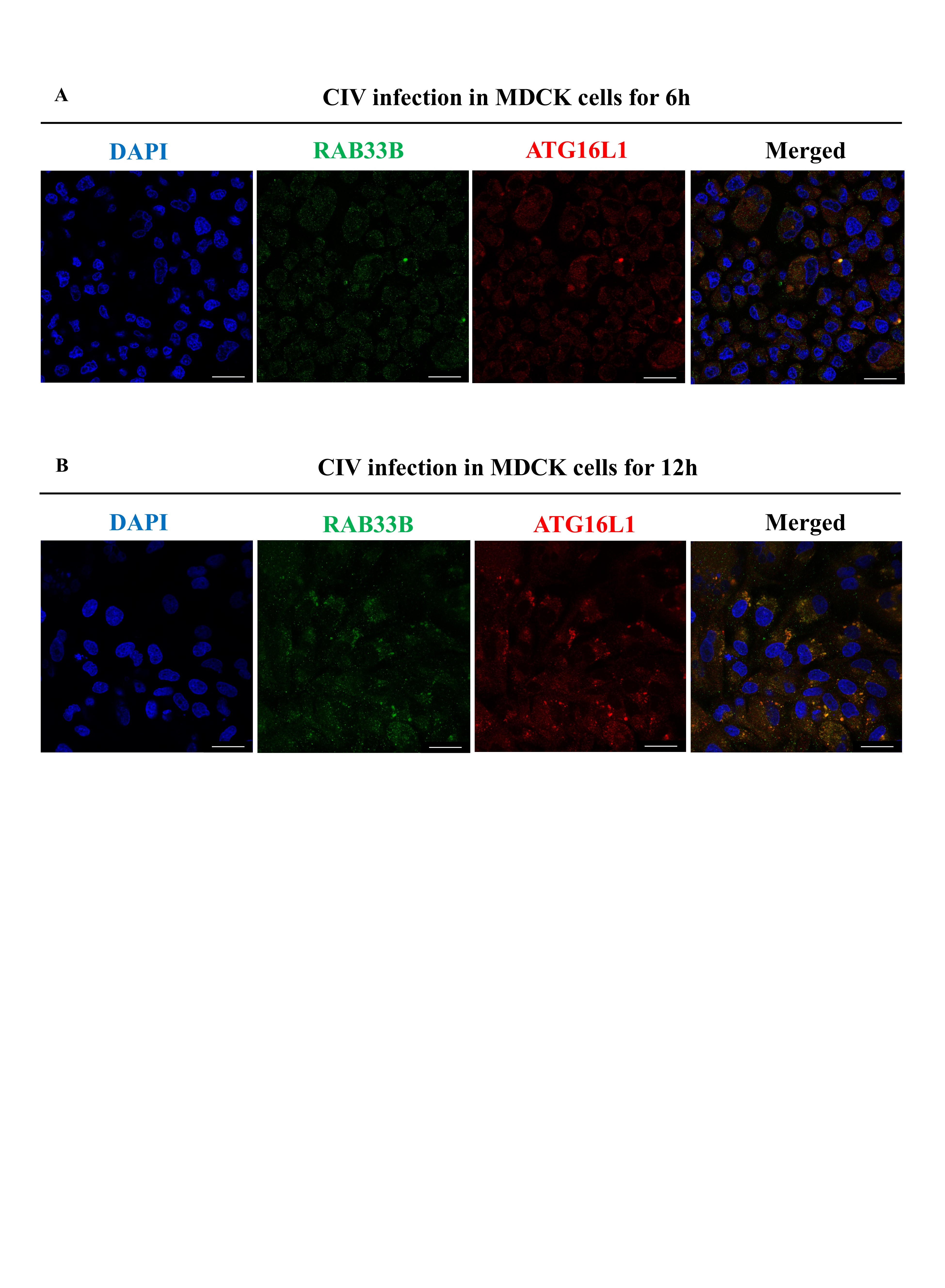

Supplement: Supplementary file 7 — Additional file 7. CIV infection activates and redistributes RAB33B and ATG16L1 protein. MDCK cells were infected with CIV at MOI = 0.1 for 6 h and 12 h, respectively. Cells were analysed for the co-localisation of RAB33B and ATG16L1. Scale bar: 20 μm. [file 13567_2025_1560_MOESM7_ESM.jpg]

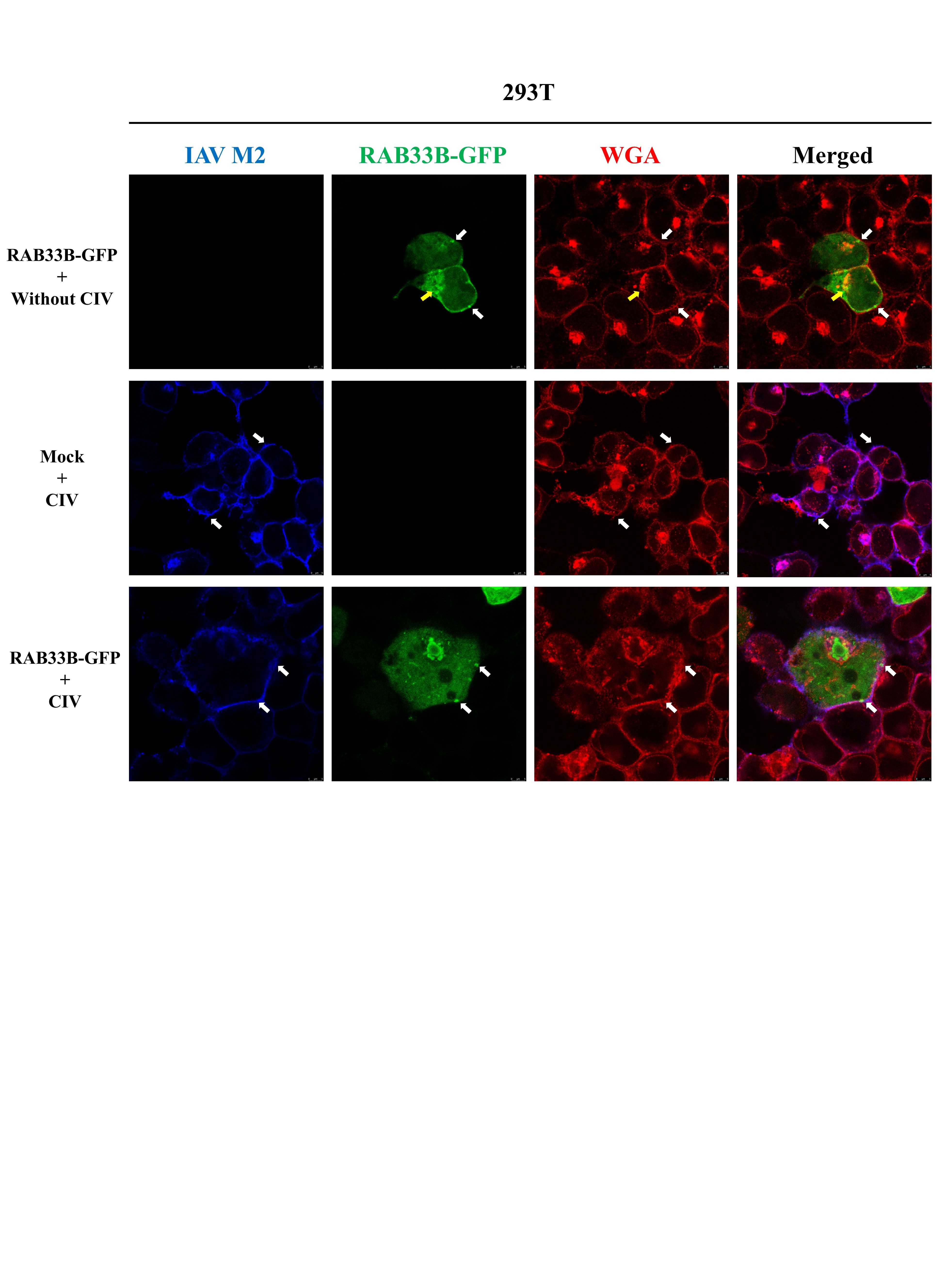

Supplement: Supplementary file 13 — Additional file 13. RAB33B-positive vesicles are localised on the M2-positive plasma membrane’s inner side. The co-localisation fluorescence indicated that RAB33B protein-positive vesicles were localised on the M2-positive plasma membrane’s inner side. WGA was used to stain the plasma membrane. The white arrow indicates the fluorescence signal on the plasma membrane’s inner side. The yellow arrow indicates the fluorescence signal around the nucleus. Scale bar: 5 μm. [file 13567_2025_1560_MOESM13_ESM.jpg]

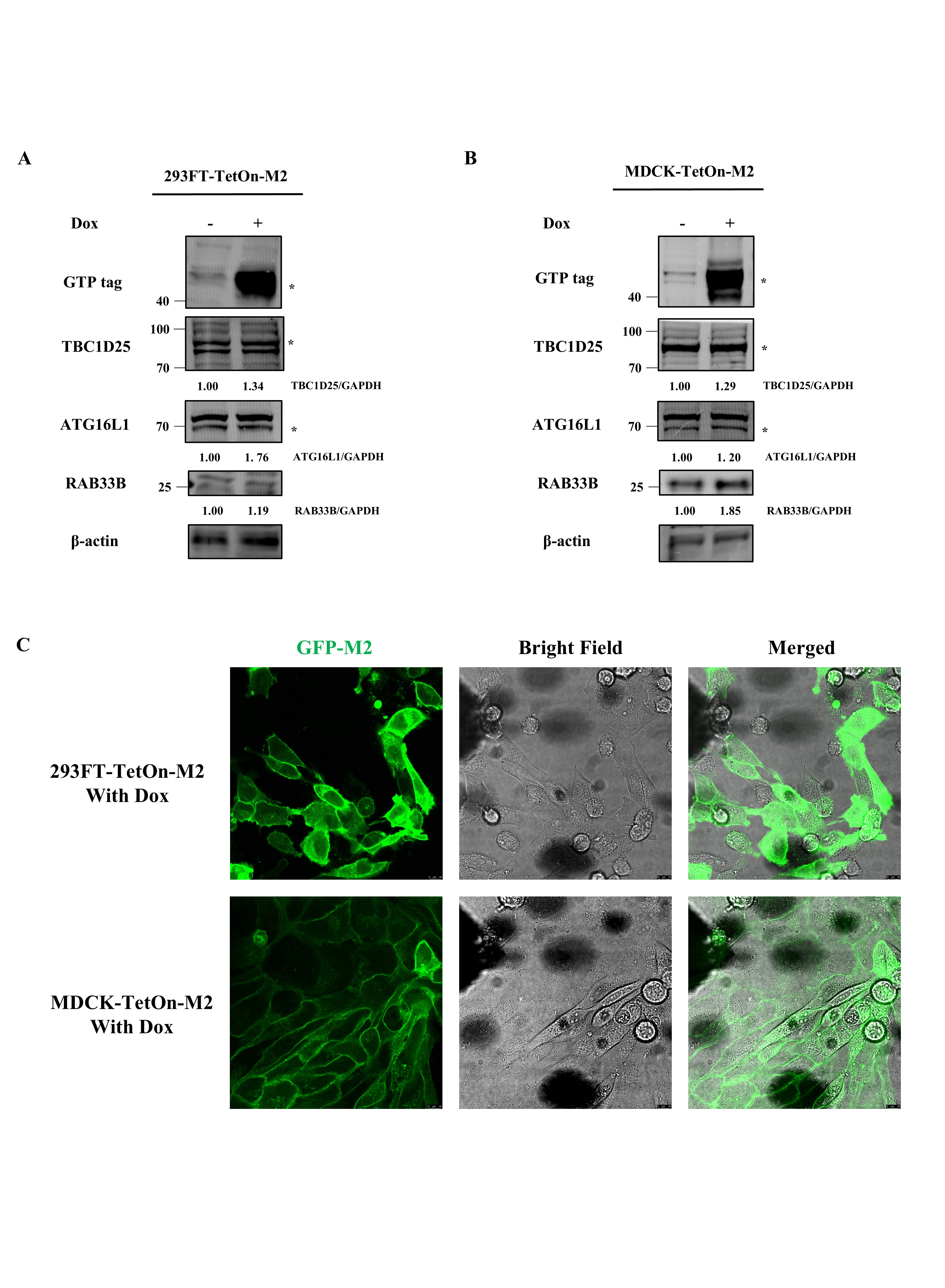

Supplement: Supplementary file 14 — Additional file 14. The establishment of HEK293FT/MDCK-TetOn-M2 cell line. A HEK293FT-TetOn-M2 cells were activated to express CIV M2-GFP by doxycycline for 48 h. Cell lysates were analysed by western blotting. * represents the indicated protein. B MDCK-TetOn-M2 cells were activated to express CIV M2-GFP by doxycycline for 48 h. Cell lysates were analysed by western blotting. * represents the indicated protein. C The stable expression of M2-GFP induced by doxycycline was confirmed. Scale bar: 10 μm. [file 13567_2025_1560_MOESM14_ESM.jpg]
