# Supplementary material for: Host cellular protein RAB33B facilitates influenza viral replication and modulates M2 trafficking by enhancing autophagy
Source: Vet Res. 2025 Jul 1;56:129. doi: 10.1186/s13567-025-01560-6 (PMC12219998; doi:10.1186/s13567-025-01560-6)
Supplement: Supplementary file 8 — Additional file 8. Plasmids used in this study. [file 13567_2025_1560_MOESM8_ESM.docx]

**Additional files 8 Plasmids used in this study.**

| **Plasmid** | **Reference** |
| --- | --- |
| CIV-M2-Myc | doi.org/10.3390/v13102048 |
| CIV-M2-N-terminal-Myc | doi.org/10.3390/v13102048 |
| CIV-M2-Central-Myc | doi.org/10.3390/v13102048 |
| CIV-M2-C-terminal-Myc | doi.org/10.3390/v13102048 |
| H1N1-M2-Myc | doi.org/10.1016/j.vetmic.2021.108997 |
| H5N1-M2-Myc | doi.org/10.3389/fmicb.2018.00303 |
| RAB33B-HA | doi.org/10.1091/mbc.e07-12-1231 |
| RAB33B-GFP | doi.org/10.1091/mbc.e07-12-1231 |
| RAB33B-BFP | doi.org/10.1091/mbc.e07-12-1231 |
| LC3-GFP-mCherry  TBC1D25-HA  TBC1D25-mCherry  TetOn-EGFP-M2 | doi.org/10.1080/15548627.2020.1725375  doi/10.1083/jcb.201008107  doi/10.1083/jcb.201008107  doi/10.1016/j.celrep.2021.109899 |
