# Supplementary material for: Host cellular protein RAB33B facilitates influenza viral replication and modulates M2 trafficking by enhancing autophagy
Source: Vet Res. 2025 Jul 1;56:129. doi: 10.1186/s13567-025-01560-6 (PMC12219998; doi:10.1186/s13567-025-01560-6)
Supplement: Supplementary file 9 — Additional file 9. Primers used in this study. Primer sets used for qPCR in this study. [file 13567_2025_1560_MOESM9_ESM.docx]

**Additional file 9** **Primer sets used for qPCR in this study.**

| **Primer** | **Sequence of oligonucleotides (5′ → 3)** | **Gene accession** |
| --- | --- | --- |
| CIV-M | TGATCCTCTCGTTATTGCCGCAAG | JX195351 |
|  | CACTCTGCTGTTCCTGCCGATAC |  |
| CIV-NP | AATAGATCCTTTCCGTCTGCTT | JX195348 |
|  | ATGCCATCCACACTAACTGACT |  |
| RAB33B | AGAACGAGCGGTGGAGATT | NM_031296 |
|  | CCATGCTCTTTCTGAATCGTT |  |
| TBC1D25 | CCAGCTCTCCCGACCCGA | NM_002536 |
|  | ATGTAGTCCATCCGCTCTCGGC |  |
| GAPDH | GAAGGTGAAGGTCGGAGTC | NM_031296 |
|  | GAAGATGGTGATGGGATTTC |  |
