# Supplementary material for: Host cellular protein RAB33B facilitates influenza viral replication and modulates M2 trafficking by enhancing autophagy
Source: Vet Res. 2025 Jul 1;56:129. doi: 10.1186/s13567-025-01560-6 (PMC12219998; doi:10.1186/s13567-025-01560-6)
Supplement: Supplementary file 10 — Additional file 10. siRNA sequences used in this study. Sequence of siRNA used in this study. [file 13567_2025_1560_MOESM10_ESM.docx]

**Additional file 10** **Sequence of siRNA used in this study.**

| **Primer** | **Sequence of oligonucleotides (5′ → 3)** |
| --- | --- |
| siRAB33B-1 | GCTAGCCAATGATATACCA |
| siRAB33B-2 | GCATGGTTCAGCACTACTA |
| siRAB33B-3 | GACCTTGGCTCATAAGCTT |
| siTBC1D25 | GGAGCCCTCGCTGCGAAAG |
